# Beyond the two-conformer model: boat conformers provide stereoselectivity in S_N_1-type glycosylations of *manno*-type donors

**DOI:** 10.1039/d6sc02312f

**Published:** 2026-04-17

**Authors:** Wouter A. Remmerswaal, Daan Hoogers, Joeri Schoenmakers, F. Matthias Bickelhaupt, Thomas Hansen, Jeroen D. C. Codée

**Affiliations:** a Leiden University, Leiden Institute of Chemistry Einsteinweg 55 2333 CC Leiden The Netherlands jcodee@chem.leidenuniv.nl; b Department of Chemistry and Pharmaceutical Sciences, Amsterdam Institute of Molecular and Life Sciences (AIMMS), Vrije Universiteit Amsterdam De Boelelaan 1108 1081 HZ Amsterdam The Netherlands; c Institute for Molecules and Materials, Radboud University Heyendaalseweg 135 6525 AJ Nijmegen The Netherlands; d Department of Chemical Sciences, University of Johannesburg Auckland Park Johannesburg 2006 South Africa

## Abstract

Oxocarbenium ions play a central role in shaping the stereochemical outcome of S_N_1-type glycosylation reactions. Generally, glycosylations involving ^4^*H*_3_-like glycosyl cations proceed with α-selectivity, whereas those involving ^3^*H*_4_-like cations furnish β-products, reflecting favorable chair-like transition states. While this analysis holds for many glycosyl cations, it breaks down for mannosyl donors. Although the mannosyl ^3^*H*_4_ cation is significantly more stable than its ^4^*H*_3_ counterpart, addition of weak carbon nucleophiles predominantly yields α-products. To elucidate the origin of this deviation from the predictive two-conformer model, we examined *C*-allylation reactions of nucleophiles spanning three orders of magnitude in reactivity with a series of glucosyl and mannosyl-type donors (mannose, rhamnose, and mannuronic acid). Quantum chemical calculations of the competing reaction pathways show that, for mannose, glycosylation proceeds under Curtin–Hammett control *via* α-attack on a *B*_2,5_-like (boat) oxocarbenium ion through an ^O^*S*_2_-type transition state that avoids the severe steric (Pauli) repulsion present along the β-^1^*C*_4_ trajectory. Activation-strain and energy-decomposition analyses quantify the steric and electronic effects and explain why rhamnose, with reduced C6 steric demand, provides slightly more β-selective glycosylation reactions and mannuronic acid, of which the ^3^*H*_4_ is exceptionally favorable, shows β-selectivity. The mechanistic framework provides a quantitative basis for understanding and designing stereoselective S_N_1-type glycosylations.

## Abstract 2

While the S_N_1 glycosylation reaction mechanism manifold is dominated by addition reactions to half chair oxocarbenium ions we here reveal that mannosyl oxocarbenium ions react on the bottom face of a boat conformer to avoid steric (Pauli) repulsion.

## Graphical abstract

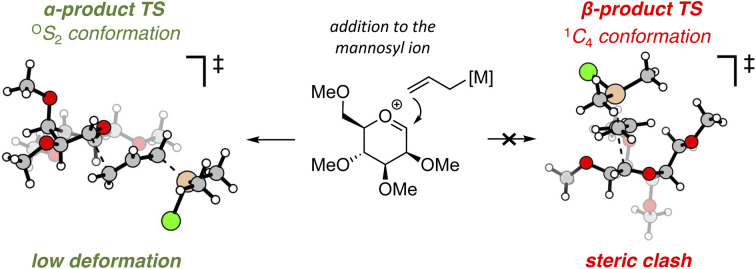

## Introduction

The structure of oxocarbenium ions plays a central role in shaping the stereochemical outcome of glycosylation reactions.^4–8^ These intermediates typically arise in S_N_1-type substitution reactions, where ionization and expulsion of the leaving group is the rate-determining step, to generate a short-lived, oxocarbenium ion that subsequently undergoes nucleophilic attack. Despite the high reactivity of these intermediates, and the fact that nucleophilic addition occurs after the rate-determining step, they can display striking stereoselectivity.^9–13^

The fleeting nature of oxocarbenium ions makes them difficult to study directly, complicating the establishment of clear structure–reactivity–stereoselectivity relationships.^14,15^ They have been studied in super-acid media, in which their lifetime is extended to allow NMR spectroscopic structural analysis,^16,17^ and *in vacuo* using IR techniques.^18–22^ Their involvement in glycosylation reactions has also been shown using kinetic isotope effect measurements^23–25^ and cation clock experiments.^26^ Quantum chemical calculations have been instrumental in providing insight into the stereoelectronic effects of the ring substituents on the structure, stability, and reactivity of these ions.^27–34^

**Fig. 1:**
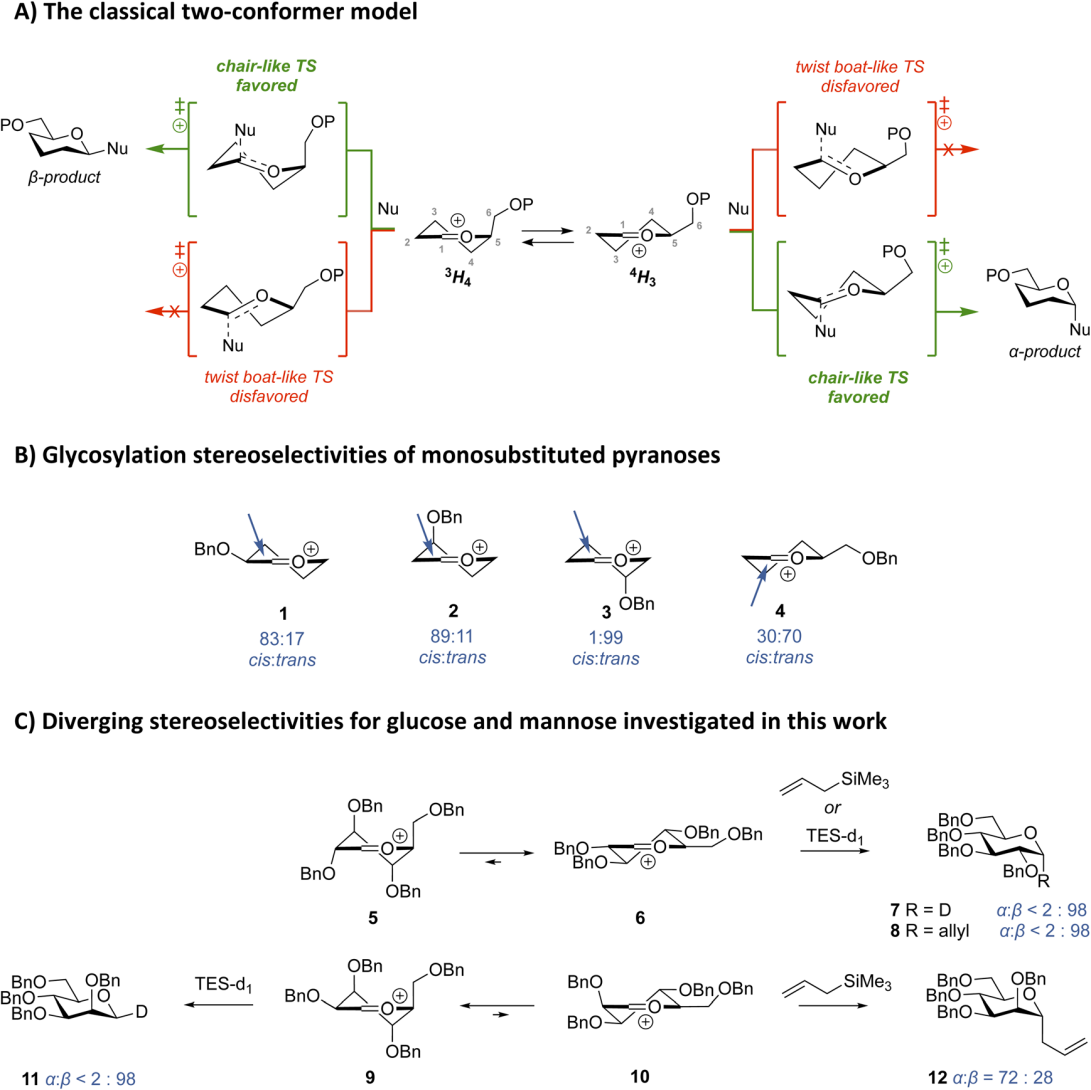
(A) The two-conformer model for understanding and predicting the stereoselectivity of glycosyl cations. P = protecting group. (B) Stereoselectivities of S_E_2′ glycosylation reactions with monosubstituted pyranosyl donors performed by Woerpel and co-workers. (C) The diverging stereoselectivities of the glycosylation reactions of the glucosyl and mannosyl cations with TES-*d*_1_ and allyltrimethylsilane investigated in this work.

In glycosylation reactions proceeding through an oxocarbenium ion intermediate, the stereochemical outcome can be rationalized by the two-conformer model (see Fig. 1A).^35,36^ This model proposes that oxocarbenium ions typically adopt one of two half-chair conformations (^3^*H*_4_ or ^4^*H*_3_) each leading to a preferred product *via* a chair-like transition state.^37,38^ The model argues that ^3^*H*_4_-like ions will undergo top-face attack to give the β-anomer, while ^4^*H*_3_-like ions undergo bottom-face attack to give the α-anomer. This relationship has been validated for many systems.^34^ Woerpel and co-workers have systematically mapped the effect of (electron withdrawing) ring substituents on the stability and reactivity of the half chair conformations and shown that the ground state conformational preference of the ions can be an all-important factor in shaping the stereochemical outcome of addition reactions on these ions.^39–43^ They showed that alkoxy substituents at C3 and C4 are most stabilizing/least destabilizing when positioned in a pseudo-axial orientation, while alkoxy -groups at C2 are placed preferentially in a pseudo-equatorial position to allow hyperconjugative stabilization of the oxocarbenium ion by the axial C2–H2 bond.^44^ On steric grounds, the C5 substituent prefers a pseudo-equatorial orientation.^45^ These orientational preferences can translate to highly stereoselective addition reactions (Fig. 1B), with the 3-*O*-benzyl substituted pyranosyl ion 2 stereoselectively delivering 1,3-*cis* addition products, while the 4-*O*-benzyl regioisomer 3 can give exclusively 1,4-*trans* products and a 2-*O*-benzyl substituent (as in 1) steers the addition reactions to favor the 1,2-*cis* products. In contrast to its conformational preference, the presence of a single C-5 alkoxymethyl group (as in 4) provides only modest selectivity towards the formation of the 1,5-*trans* product, highlighting the subtleties involved even in simple systems.^39^

When multiple substituents are present their effects can be mutually supportive, or they can counteract one another. To gauge the overall effect of the complete substitution pattern, we have previously developed computational methods to study the structure and stability of fully substituted glycosyl oxocarbenium ions and mapped how the ring substituents affect the overall shape of the ions,^34,46^ and methodologies to compare the addition reactions.^47^ The striking *cis*-selectivity in addition reactions to many pyranosyl oxocarbenium ions could be explained from the ground state preference of the involved ions. For example, for the glucosyl ion the ^4^*H*_3_ ion 6 was found to be the most favorable oxocarbenium ion conformer, and addition reaction of triethylsilane-*d* (TES-*d*_1_) provided the 1,2-*cis* (α) product 7 with exclusive stereoselectivity (Fig. 1C). The addition reaction of TES-*d*_1_ to the mannosyl cation, which preferentially takes up a ^3^*H*_4_ conformation (9), also proceeds with exclusive 1,2-*cis*-stereoselectivity to deliver the β-product 11 (Fig. 1C).^34^

However, the nature and reactivity of the nucleophile can have a strong effect on the stereochemical course of the addition reactions. When weaker and/or more sterically demanding nucleophiles are used, the stereoselectivity of the addition reactions can change because interactions between the incoming nucleophile and oxocarbenium ion become important. The mannosyl oxocarbenium ion presents a prominent example: where the addition of TES-*d*_1_ stereoselectively provides the β-product, the corresponding *C*-allyl mannoside 12 is formed with (moderate) α-selectivity when allyltrimethylsilane is used as a nucleophile.^45^

Woerpel and co-workers have proposed that the erosion of stereoselectivity in this case is caused by the steric interactions of the incoming nucleophile and the most stable ^3^*H*_4_ mannosyl oxocarbenium ion 9. By invoking a Curtin–Hammett kinetic scenario, they reasoned that the major α-product is formed from the least populated ^4^*H*_3_ conformer 10, which is in rapid equilibrium with its more stable ^3^*H*_4_ counterpart. This change in stereoselectivity is not observed for the glucosyl ion, because the incoming nucleophile does not encounter prohibitively large steric interactions on the α-face of the ^3^*H*_4_ ion 6.

To unravel the origin behind the diverging reaction outcomes and understand why and when different Curtin–Hammett scenarios come into play in addition reactions of the mannosyl oxocarbenium ion – and by extension other glycosyl cations – we have investigated a systematic set of *C*-allylation reactions in which we have paired different allyl nucleophiles of gradually changing reactivity with gluco- and *manno*-configured donors. To understand the stereoelectronic effects that shape the Curtin–Hammett kinetic scenario in the addition reactions to the mannose oxocarbenium ion, we have probed mannosyl, rhamnosyl and mannuronic acid systems. These have revealed clear reactivity-stereoselectivity trends. We have employed quantum chemical calculations, including potential energy surface (PES) mapping and activation strain model (ASM) analyses, to investigate the different reaction paths. These studies have identified an unexpected low-energy pathway for the addition reactions to the mannosyl cation. The steric interactions in the transition state of the addition reaction taking place on the β-face of the ^3^*H*_4_ mannosyl cation and relatively high energy of the ^4^*H*_3_ intermediate make that an alternative pathway, involving α-attack on a boat-like (*B*_2,5_) intermediate, becomes most favorable. Our ASM analyses have quantified the steric and electronic effects in the different transition states, providing quantitative insight into how they steer the reaction outcome.

## Results and discussion

### Model glycosylation reactions

To investigate the different addition pathways on glycosyl oxocarbenium ions, we examined glucosyl and mannosyl donors 13 and 14 (Table 1). Each of these was reacted with a series of allyl nucleophiles of similar size but spanning three orders of magnitude in nucleophilicity: allyl (chloro)dimethylsilane (*N* = −0.57), allyltrimethylsilane (*N* = 1.68), and allyltributylstannane (*N* = 5.45).^48–50^ The results of these *C*-allylation reactions are summarized in Table 1. The glucosyl donor 13 predominantly delivered the α-products with all nucleophiles tested. The erosion of α-selectivity observed with allyltributylstannane may be accounted for by the higher reactivity of this nucleophile, making direct substitution of the intermediate α-triflate through a more associative S_N_2-like transition state possible. The stereoselectivity of the reactions of the mannosyl donor 18 exhibited a much larger dependence on nucleophile reactivity. The reaction with the weakest allyl nucleophile, showed complete α-selectivity (α : β > 98 : 2), which shifted to a moderate α-selectivity (α : β = 72 : 28) for allyltrimethylsilane (in line with reports by Crich^51^), to a moderately β-selective reaction (28 : 72) for the strongest nucleophile (in line with reports by McGarvey^52^). The results for the weaker nucleophiles, allyl (chloro)dimethylsilane and allyltrimethylsilane, may be accounted for by Woepel's hypothesis that the α-products are formed from the minor ^4^H_3_-like oxocarbenium ion (10), that is in equilibrium with its more stable ^3^*H*_4_-counterpart (9). In the chair-like transition state originating from the ^3^*H*_4_-oxocarbenium ion, steric interactions will develop with the pseudo-axial substituents at C3 and C5. With weaker nucleophiles, that react through a later transition state these interactions will be more severe, making this transition state less favorable. The higher α-selectivity for the weakest nucleophile (allyl (chloro)dimethylsilane) in comparison with allyltrimethylsilane is in line with this hypothesis. An earlier transition state for allyltributylstannane, (partially) restores the β-selectivity, predicted by the two-conformer model. A more associative pathway, in which the anomeric α-triflate (or contact ion pair) is substituted by the nucleophile can also contribute to the 1,2-*cis* selectivity.

**Table 1:** Model *C*-glycosylation reaction results from glucosyl donor, 13, and mannosyl donor, 14. The stereoselectivity of the reaction is expressed as α : β and based on ^1^H-NMR of purified α/β-product mixtures. Pre-activation-based glycosylation conditions were used: donor 13 or 14 (1.0 eq.), Tf_2_O (1.3 eq.), Ph_2_SO (1.3 eq.), TTBP (2.5 eq.), DCM (0.05 M), −80 to −60 °C, then add nucleophile (2.0 eq.) at −80 °C and allow to warm to −60 °C^1^

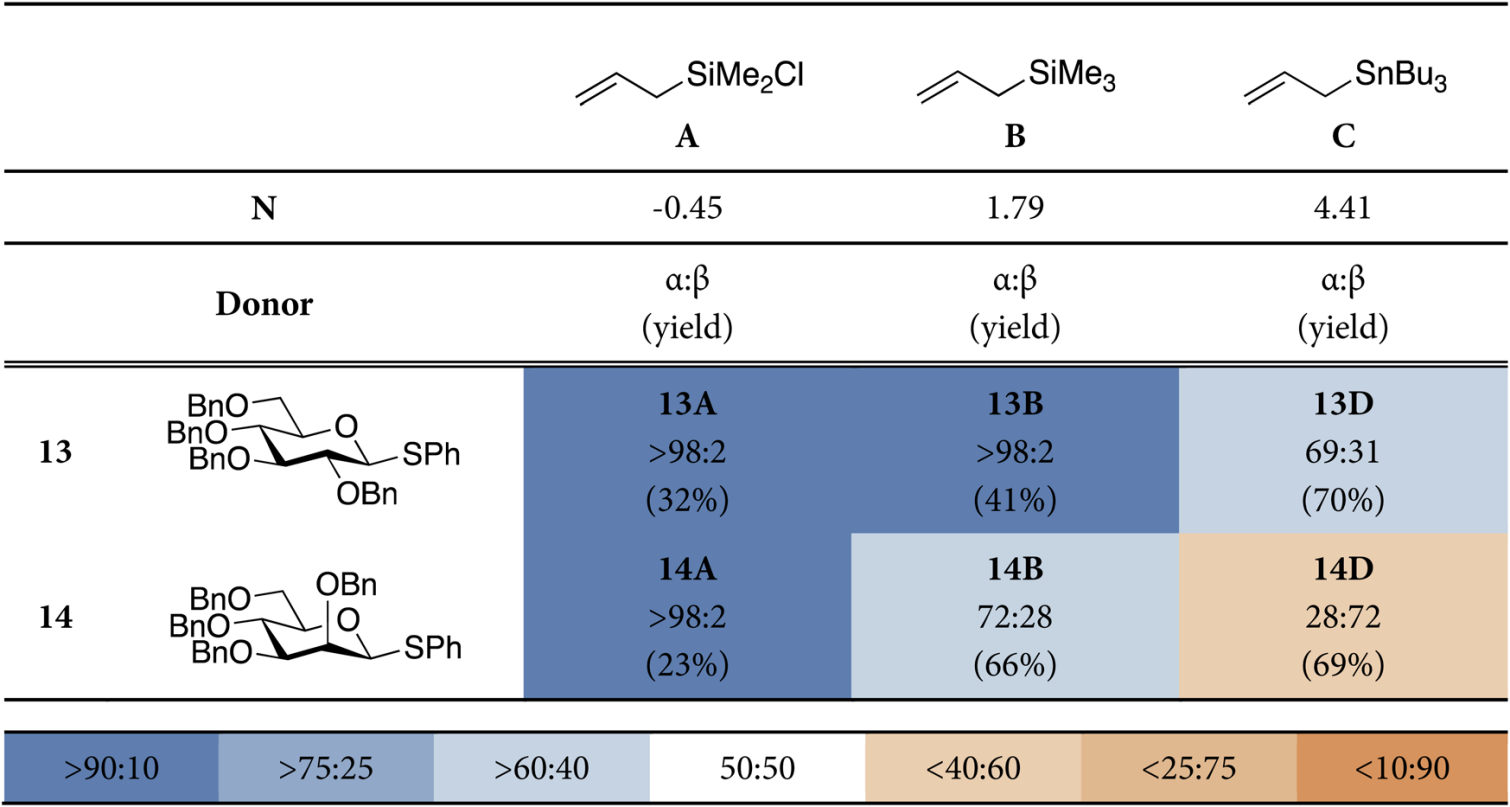

### Computational analysis

To better rationalize these experimental observations, we computed potential energy surfaces for these addition reactions in DCM solution at the PCM(CH_2_Cl_2_)-B3LYP/6-311G (d,p) level of theory. Benzyl groups were replaced with methyl ethers to reduce computational cost. In several cases, the electronic potential energy surface for reactions with these cationic species appeared barrierless. However, entropic contributions often introduced an overall reaction barrier. To identify the corresponding barriers, we performed constrained scans along the reaction coordinate and evaluated partition functions at each computed point to obtain Gibbs free energies. This approach was benchmarked against systems for which both constrained and unconstrained reaction barrier were available (see SI for all computational details) and was found to reproduce consistent trends.

**Fig. 2:**
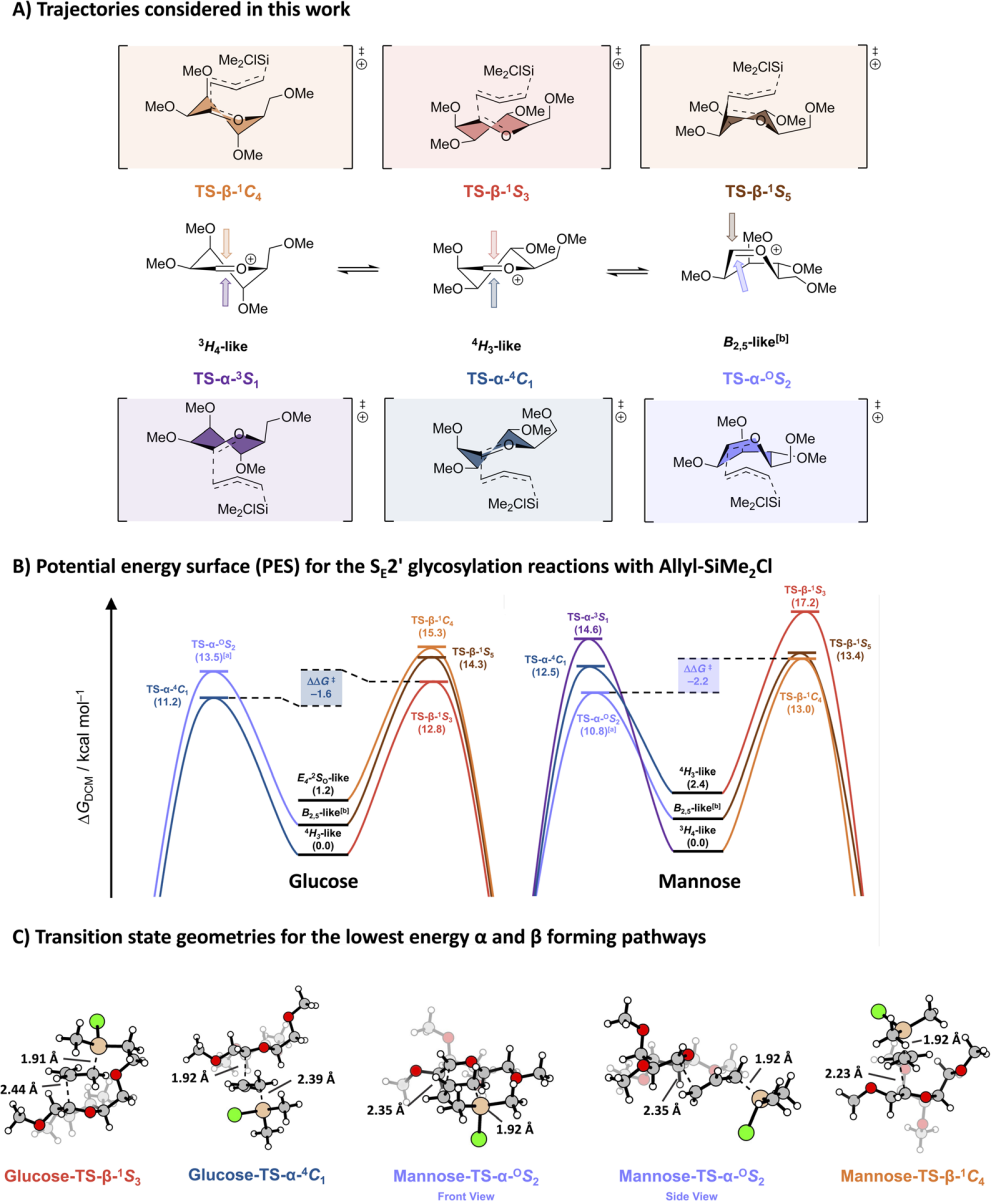
(A) An overview of the possible S_E_2′ reaction trajectories considered in this work, and (B) the corresponding reaction profiles of the addition of allyl (chloro)dimethylsilane to the glucosyl and mannosyl cations *via* the following transition states: TS-β-^1^*C*_4_ (orange), TS-β-^1^*S*_5_ (brown), TS-β-^1^*S*_3_ (red), TS-α-^O^*S*_2_ (light violet), TS-α-^4^*C*_1_ (blue), and TS-α-^3^*S*_1_ (purple). Energies are depicted as Gibbs free energies (*T* = 213.15 K) and were computed at PCM(CH_2_Cl_2_)-B3LYP/6-311G (d,p). The TS-α-^3^*S*_1_ on glucose is unfavourable and could not be located. ^[*a*]^A representative structure for the transition state geometry was used.^2^^[*b*]^The itinerary leading up to TS-α-^O^*S*_2_ and TS-β-^1^*S*_5_ start from a boat-like non-stationary point. (C) Structures of the lowest energy transition states of the S_E_2′ reactions of ally (chloro)dimethylsilane with the glucosyl or mannosyl cations leading to the α and β products. The mannose-TS-α-^O^*S*_2_ is depicted from two distinct angles to show the ‘sideways’ approach of the nucleophile, avoiding most steric interaction.

For the most nucleophilic allylic reagents, such as allyltributylstannane, we were unable to locate transition states on the electronic potential energy surface. As discussed above, the high reactivity of these nucleophiles leads to barrierless additions. Because the strongest deviation from the stereoselectivity predicted by the two-conformer model was found in the additions of allyl (chloro)dimethylsilane, and this nucleophile also allowed for the location of most transition states, these will be used in the following analyses and discussion. For each donor-nucleophile pair, multiple plausible trajectories were identified: α-attack *via* a chair-like ^4^*C*_1_ from a ^4^*H*_3_, a ^3^*S*_1_ from a ^3^*H*_4_, or an ^O^*S*_2_ from a boat-like *B*_2,5_; and β-attack *via* a ^1^*C*_4_ from a ^3^*H*_4_, a ^1^*S*_3_ from a ^4^*H*_3_, or a ^1^*S*_5_ from a *B*_2,5_ (Fig. 2A). Fig. 2B compares the barrier heights for the addition reactions of allyl (chloro)dimethylsilane on the glucosyl and mannosyl cations. The ΔΔ*G*^‡^ values of −1.6 kcal mol^−1^ (glucose) and −2.2 kcal mol^−1^ (mannose) between the lowest α- and β-forming transition states are fully consistent with the experimentally observed >98 : 2 α-selectivities for allyl (chloro)dimethylsilane: application of the Eyring equation at −60 °C (213 K) gives predicted α : β ratios of *ca.* 98 : 2 and 99.5 : 0.5, respectively.

For the glucosyl cation, the lowest transition state (the ^4^*C*_1_-like TS, Δ*G* = 11.2 kcal mol^−1^) originated from the lowest energy oxocarbenium ion half chair (the ^4^*H*_3_-ion 6), in keeping with the two-conformer model. As previously established, the latter half chair is more stable than its counterpart on the other side of the Cremer-Pople conformational sphere, which adopts a the *E*_4_/^2^*S*_O_ -like structure. This conformer deviates from the canonical ^3^*H*_4_ conformation (5, shown in Fig. 1) to diminish the steric interactions between all the pseudo-axial substituents in this half chair conformation. These steric interactions also make the transition states departing from this conformer unfavorable. As a result, attack of the nucleophile on the β-face of the lowest energy ^4^*H*_3_ ion, which deforms the glucosyl cation into a ^1^*S*_3_-like structure in the transition state, represents the lowest energy pathway to the β-products, being more favorable than the chair-like transition state originating from the *E*_4_/^2^*S*_O_ -like ion.

The mannosyl ^3^*H*_4_ cation 9 is significantly more favorable than its ^4^*H*_3_ counterpart 10 (by 2.4 kcal mol^−1^). However, the trajectory of the incoming nucleophile on the β-face of the former ion (see Fig. 2C, mannose-TS-β-^1^C_4_), is met with significant steric interactions, which makes this pathway rather unfavorable (13.0 kcal mol^−1^). The chair-like transition state originating from the alternative, higher energy ^4^*H*_3_ half-chair oxocarbenium ion 10 was found to be more favorable (12.5 kcal mol^−1^). Notably the most favorable approach we located, involved attack of allyl (chloro)dimethylsilane on the α-face of the *B*_2,5_ conformer of mannose. In this mode of attack the mannose ring adopts a ^O^*S*_2_-like conformation, thereby avoiding severe steric interactions between the nucleophile and pseudo-axial C3 and C5 substituents, which destabilize the β-^1^*C*_4_ approach (see Fig. 2C, mannose-TS-α-^O^*S*_2_). Notably, Crich and co-workers have previously described a similar transition state conformation for the intramolecular attack of an C2-trimethylsilyl (methallyl) ether in a benzylidene mannose system.^22^ We could also find a transition state for the attack of the nucleophile on the β-face of the *B*_2,5_ mannosyl ion, but this was found to be significantly less favorable (13.4 kcal mol^−1^).

While the potential energy surfaces and transition state barriers discussed above are reported as Gibbs free energies (Δ*G*), we verified that the relative reactivity trends are identical when considering the corresponding electronic energies (Δ*E*). Accordingly, the selectivity trends observed in the PES analysis can be rationalized using the activation strain model (ASM) of reactivity and energy decomposition analysis (EDA), which describe the reaction profile in terms of electronic energies.^53–56^ In this fragment-based approach, the potential energy surface can be described with respect to, and understood in terms of, the characteristics of the reactants, here the allylic nucleophile and the glycosyl oxocarbenium ion substrate. The energy (Δ*E*) along the potential energy surface is expressed as the sum of the strain energy (Δ*E*_strain_) required to deform the individual reactants from their equilibrium geometries and the interaction energy (Δ*E*_int_) between the deformed fragments: Δ*E* = Δ*E*_strain_ + Δ*E*_int_. These are then plotted along the potential energy surfaces in activation strain diagrams (ASDs). Here, the glycosyl oxocarbenium ion and the allylic *C*-nucleophile served as the two fragments. The intrinsic reaction coordinate (IRC) was projected onto the carbon-leaving group (C⋯Si) distance of the nucleophile, which changes in a well-defined manner during the S_E_2′ attack, and this coordinate has been validated as a suitable reaction coordinate for this type of bimolecular processes.^47^

Energy decomposition analysis (EDA) was applied to the Δ*E*_int_ term to dissect it into the electrostatic interactions (Δ*V*_elstat_), Pauli repulsion (Δ*E*_Pauli_), and orbital interaction energy (Δ*E*_oi_). EDA values are reported as gas-phase electronic energies (Δ*E*_gas_).^57^ For consistent comparison, ASM and EDA profiles for different pathways were analyzed at points with identical C⋯Si distances. Because the EDA terms are highly dependent on the distance between the fragments,^55,56,58,59^ we additionally verified the results using double-consistent geometries (DCGs) in which both the C⋯Si and C1⋯C-allyl distances were matched (see SI for computational details). This eliminates geometric artefacts that can distort interaction terms, especially the Pauli repulsion term. All DCG-verified data, including geometries and numerical values, are provided in SI Fig. S5 and S6.

**Fig. 3:**
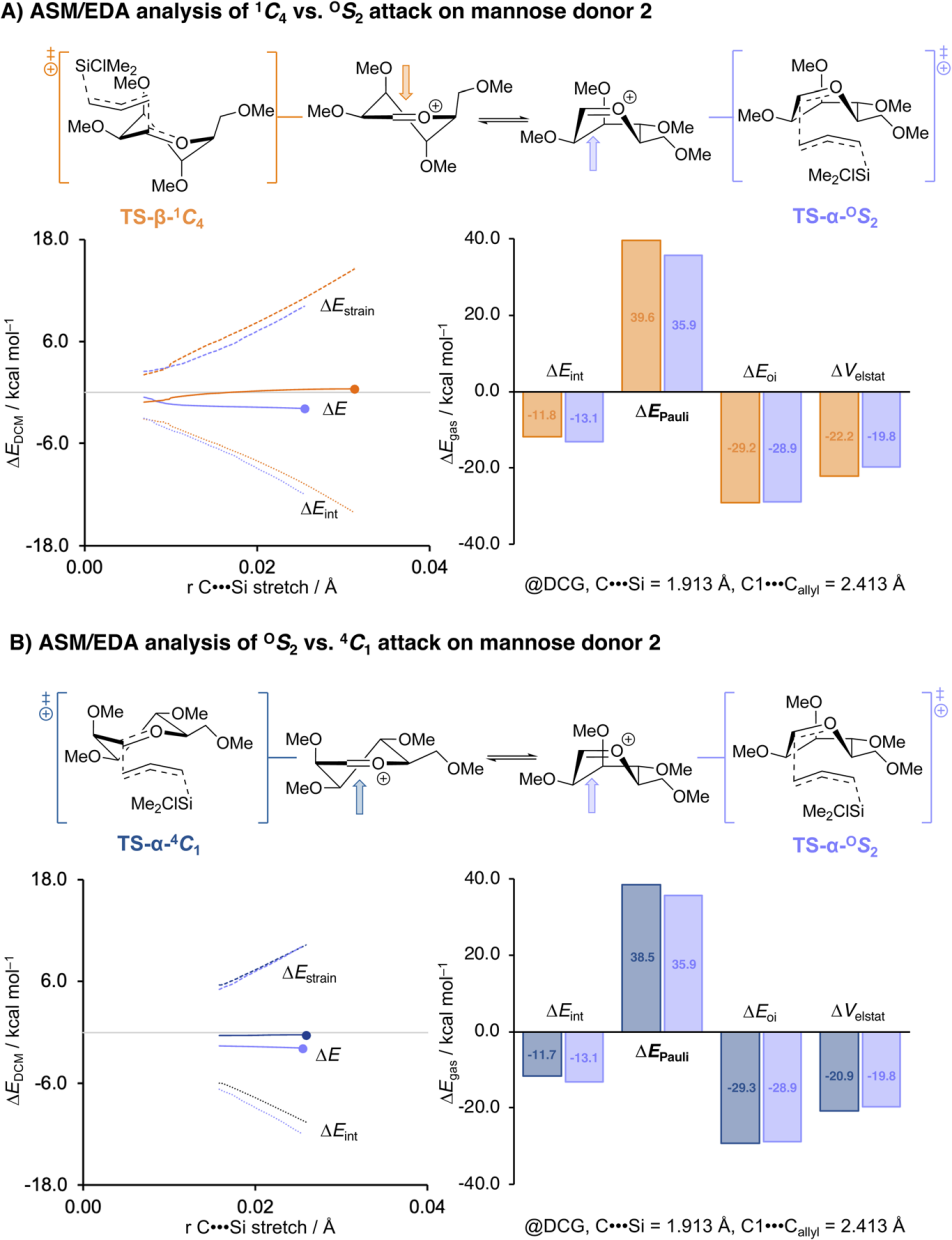
Activation strain and energy decomposition analyses for the S_E_2′ reactions of allyl (chloro)dimethylsilane and the mannosyl cation (A) *via* the β-product-forming TS-β-^1^*C*_4_ (orange) and the α-product-forming TS-α-^O^*S*_2_ (light violet) and (B) *via* both α-product-forming TS-α-^4^C_1_ (blue) and TS-α-^O^*S*_2_ (light violet). ASM energy values are plotted to the transition state (indicated by a dot), along the IRC projected on the C⋯Si bond stretch. EDA results are numerical experiments at double consistent TS-like geometries (ΔE*) obtained from the IRC at a C⋯Si bond stretch of 1.913 Å. The C1⋯C_allyl_ were set to 2.413 Å (C⋯Si and C1⋯C_allyl_ distances in the consistent TS-like geometry for TS-α-^4^*C*_1_). Energies are depicted as electronic energies and were computed at PCM(CH_2_Cl_2_)-B3LYP/6-311G (d,p) for Δ*E*_DCM_ or ZORA-B3LYP/TZ2P//PCM(CH_2_Cl_2_)-B3LYP/6-311G (d,p) for Δ*E*_gas_.^3^

Our activation strain analyses revealed that, for mannose, the β-^1^*C*_4_ pathway accumulates strain more steeply along the C⋯Si coordinate than the competing α-^O^*S*_2_ pathway (Fig. 3A, orange). This reflects the deformation required for the ^3^*H*_4_ conformer to accommodate the nucleophilic approach on the β-face, which brings the incoming nucleophile into close proximity with pseudo-axial C3 and C5 substituents. Steric congestion initially manifests as Pauli repulsion (as evident from the corresponding EDA term), part of which is converted into strain upon deformation. Although this strain remains destabilizing, it is less unfavorable than the scenario without deformation, in which Pauli repulsion would increase even more strongly. In contrast, while the α-^O^*S*_2_ pathway (light violet in Fig. 3A) starts from a higher-energy *B*_2,5_ conformer it avoids the most severe steric interactions, which results both in less strain build-up along the reaction trajectory, and less Pauli repulsion from the start of the nucleophilic approach.

Crucially, this mechanistic option is viable because the ^3^*H*_4_ and *B*_2,5_ conformers of the mannosyl cation are relatively close in energy. The small energy gap between these means that the additional strain required to reach the *B*_2,5_ geometry is not prohibitive under Curtin–Hammett control. Were the ^3^*H*_4_-*B*_2,5_ gap appreciably larger, the cost of conformer interconversion would outweigh the gain in interaction energy in the ^O^*S*_2_ transition state and the canonical β-^1^*C*_4_ pathway would be expected to be dominant. Thus, the combination of strong Pauli repulsion for β-attack on the ^3^*H*_4_ conformer and a narrow ^3^*H*_4_/*B*_2,5_ energy gap enables the boat-like itinerary to prevail over the classical two-conformer model.

Finally, it was examined why the bottom-face attack on a boat-like conformation leading to TS-α-^O^*S*_2_ is more favorable than the canonical bottom-face attack on a half chair-like conformation (TS-α-^4^*C*_1_). The ASM analyses are depicted in Fig. 3B, which show the ASDs for these TS-α-^4^*C*_1_ (blue) *versus* the TS-α-^O^*S*_2_ (light violet) pathways. From the C⋯Si bond stretch diagram, it can be seen that both bottom face pathways incur similar strain, while the approach leading to TS-α-^4^*C*_1_ is paired with less stabilizing interaction energy. The increased destabilizing Pauli repulsion for the TS-α-^4^*C*_1_ pathway is the main contributor to the observed difference in interaction energy. While this might seem counterintuitive, this indicates that at the same C_1_⋯C_allyl_ bond distance, the nucleophile is positioned more favorably in the TS-α-^O^*S*_2_ pathway, than in the TS-α-^4^*C*_1_. Inspection of the structures in Fig. 2, reveals that the terminal allyl methylene group is positioned under the mannose ring in the TS-α-^4^*C*_1_ while it approaches the cation parallel to the C=O^+^ moiety in the TS-α-^O^*S*_2_ avoiding steric interactions with the electrophile.

### C6-modified donors

To further test the relative importance of the Pauli repulsion on the β-face and the energy gap between the competing ^3^*H*_4_ and *B*_2,5_ conformers we next explored different mannosyl-configured donors, carrying different C6-modifications. We turned to rhamnose and mannuronic acid donors, in which the C6-CH_2_OBn group of mannose is replaced by the slightly smaller and electronically different CH_3_ or CO_2_Me group, respectively. Mannuronic acid donors have been shown to provide 1,2-*cis* products with a wide variety of acceptors in a very reliable manner, and it has been reported that in the *C*-glycosylation reaction with allyltrimethylsilane solely the β-allyl products are obtained,^60^ in contrast to their mannose counterparts.

**Fig. 4:**
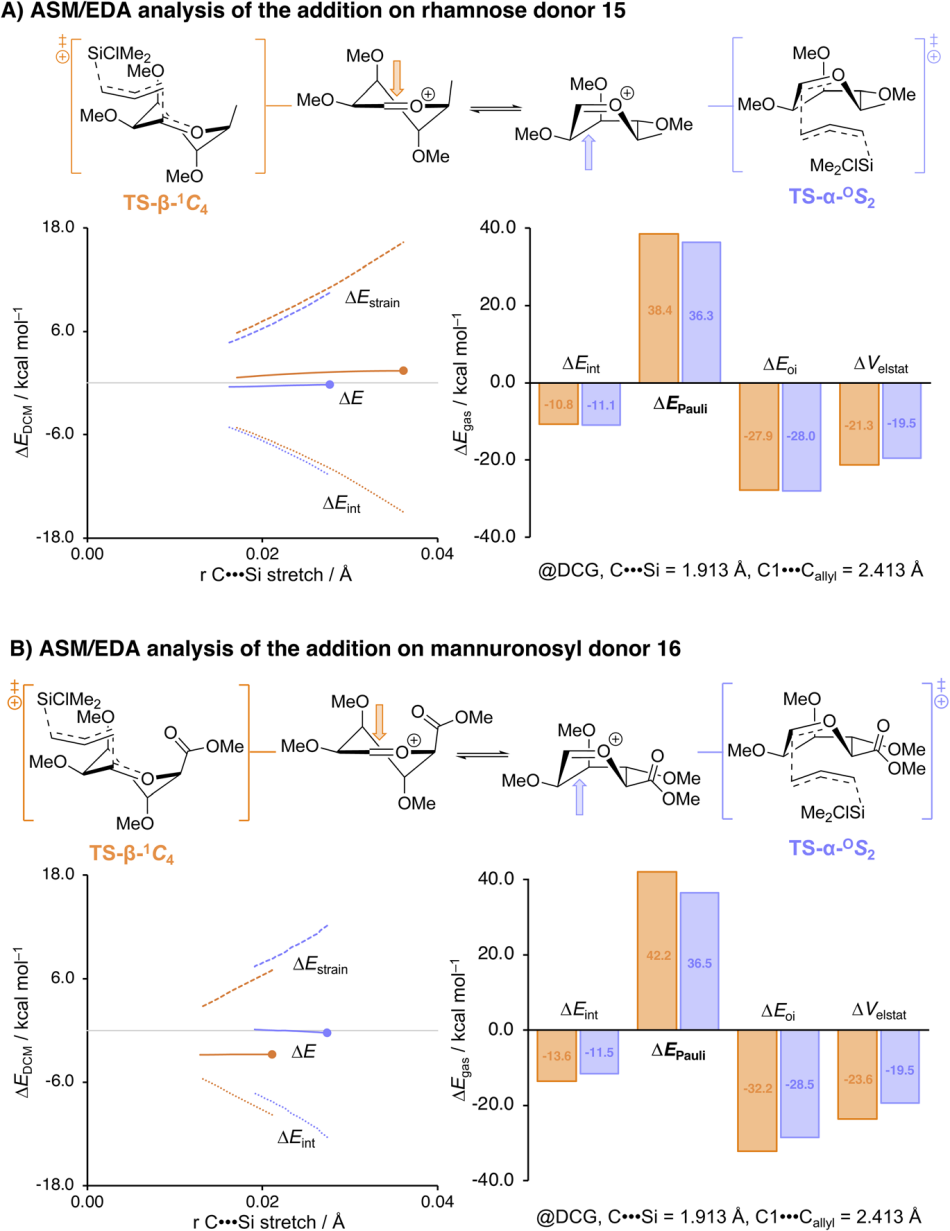
Activation strain and energy decomposition analyses for the S_E_2′ reactions of allyl (chloro)dimethylsilane and the (A) rhamnosyl cation and (B) the mannuronosyl cation *via* the β-product-forming TS-β-^1^*C*_4_ (orange) and the α-product-forming TS-α-^O^*S*_2_ (light violet). Energy values are plotted to the transition state (indicated by a dot), along the IRC projected on the C⋯Si bond stretch. Energies are depicted as electronic energies and were computed at PCM(CH_2_Cl_2_)-B3LYP/6-311G (d,p) for Δ*E*_DCM_ or ZORA-B3LYP/TZ2P//PCM(CH_2_Cl_2_)-B3LYP/6-311G (d,p) for Δ*E*_gas_.

Thus, we subjected the rhamnosyl and mannuronosyl donors, 15 and 16, respectively, to the same set of model *C*-nucleophiles, as described above, to reveal the stereoselectivity trends shown in Table 2. We also performed the ASM/EDA analyses for the addition reactions for these donor systems. The profiles generated by these computational approaches are depicted in Fig. 4. Full potential energy profiles for the addition reactions to the rhamnosyl and mannuronosyl cations are provided in the SI (SI Fig. S3 and S4).

**Table 2:** Model *C*-glycosylation reaction results from mannosyl donor, 14, rhamnosyl donor, 15, and mannuronosyl donor, 16. The stereoselectivity of the reaction is expressed as α : β and based on ^1^H-NMR of purified α/β-product mixtures. Pre-activation-based glycosylation conditions were used: donor 14–16 (1.0 eq.), Tf_2_O (1.3 eq.), Ph_2_SO (1.3 eq.), TTBP (2.5 eq.), DCM (0.05 M), −80 to −60 °C, then add nucleophile (2.0 eq.) at −80 °C and allow to warm to −60 °C^1^

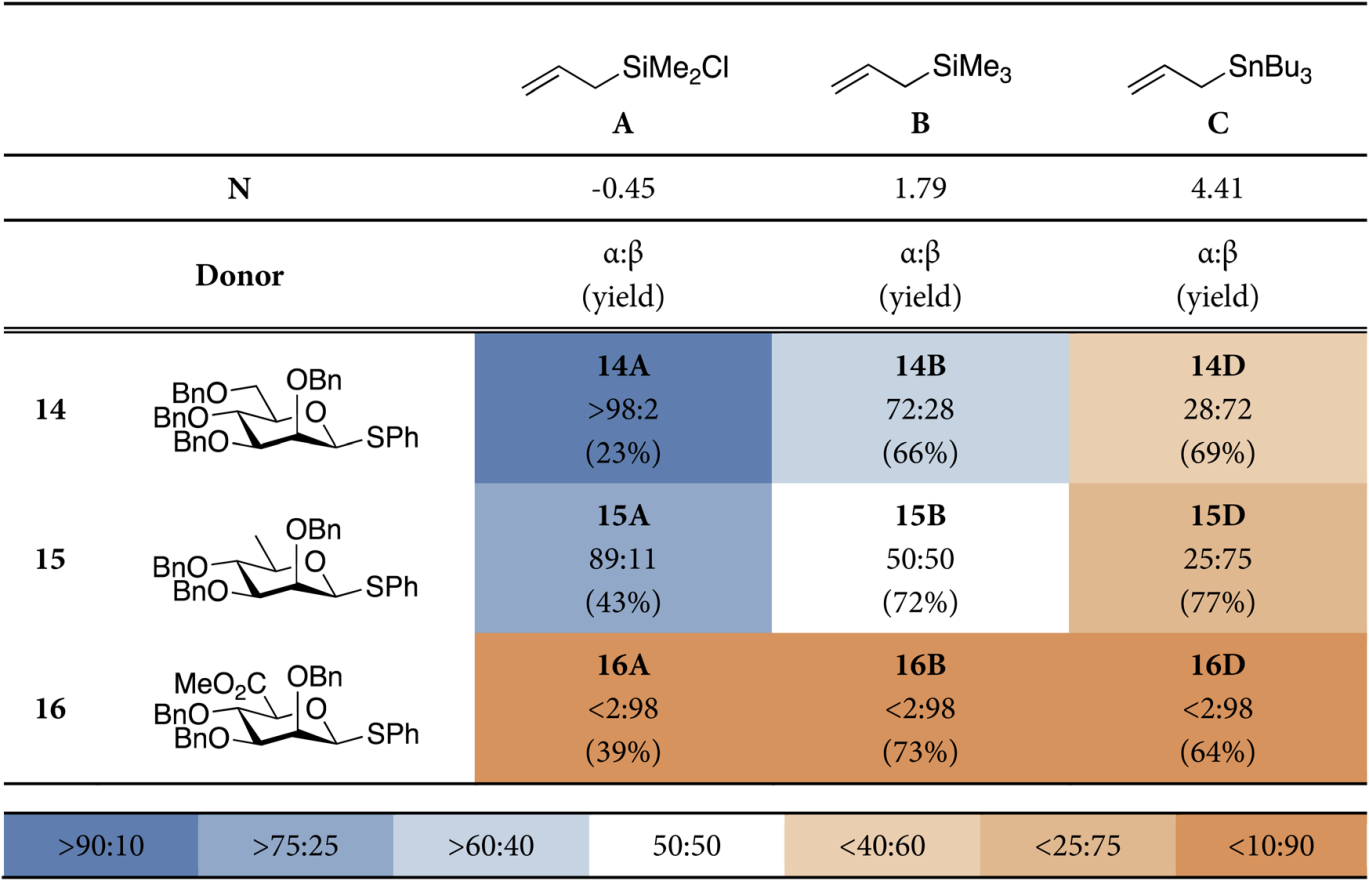

The rhamnose donor 15 displayed a reactivity-stereoselectivity trend that closely paralleled that of the mannosyl donor: across the series of allyl nucleophiles the product distribution shifted from the predominance of the α-product for the weakest nucleophile towards formation of more β-product with the stronger nucleophiles. The rhamnosylations were moderately but consistently more 1,2-*cis* selective than the mannosylations. The increase in the formation of the β-products can be rationalized by examining the ASM/EDA analyses. The reduced steric demand on the β-face delays the onset of Pauli repulsion and lowers its maximum along the β-^1^*C*_4_ trajectory, flattening the energy profiles and narrowing the energy difference between β- and α-attack (Fig. 4A). As a result, the kinetic advantage of the α-^O^*S*_2_ pathway is diminished, in comparison to the mannosylation reactions.

In sharp contrast to the reactions of the mannosyl and rhamnosyl donors, the mannuronic acid donor 16 furnished exclusively the β-products regardless of the reactivity of the used *C*-nucleophile. This behavior is consistent with an approach to the β-face of the most favorable ^3^*H*_4_-like oxocarbenium ion and in line with the predictive two-conformer model. The persistence of β-selectivity with weak nucleophiles, where α-product formation could emerge through a Curtin–Hammett scenario, can be attributed to the strong preference for the ^3^*H*_4_ oxocarbenium ion conformer. This conformer is stabilized (with respect to the other conformers) by the carboxylate ester, which prefers to occupy a (pseudo)-axial orientation in an oxocarbenium ion species. When the ASM/EDA of the addition to the mannuronic acid ester ion is regarded, the effect of the carboxylic acid ester immediately becomes apparent (Fig. 4B); the β-^1^*C*_4_ pathway is associated with both significantly less strain than the α-^O^*S*_2_ itinerary and more stabilizing interaction energy. When the interaction energy is analyzed a trend similar to the mannosyl cation (Fig. 3A) is observed, the TS-α-^O^*S*_2_ pathway proceeds with significantly less Pauli repulsion and the β-^1^*C*_4_ pathway proceeds with more stabilizing electrostatic and orbital interactions. The difference in strain is already present at the start of the pathway, which can be traced back to the stability of the ^3^*H*_4_ oxocarbenium ion. The high stability of this conformer raises the barrier to the formation of other conformers and prevents access to the *B*_2,5_-^O^*S*_2_ pathway that is the dominant addition trajectory to the mannosyl ion.

## Conclusions

This combined experimental and computational study establishes that the classical two-conformer model, while predictive for many glycosyl donors, does not adequately describe S_N_1-type glycosylations of *manno*-configured donors. Detailed potential-energy surface mapping and activation-strain/energy-decomposition analyses reveal that attack of the mannosyl cation, leads to strong steric (Pauli) repulsion along the β-^1^*C*_4_ trajectory. The small ground-state energy difference between ^3^*H*_4_ and *B*_2,5_ conformers, allows an alternative pathway to become competitive: α-attack on a *B*_2,5_-like cation that passes through an ^O^*S*_2_ transition state. This route provides a lower overall barrier than the canonical chair-like β-attack and accounts for the α-selectivity observed with weak nucleophiles.

Modifications of C6 of the mannose donor confirm and quantify the roles of steric and conformational effects. Reducing steric demand by utilizing a C6-CH_3_ group (as in rhamnose) lowers steric (Pauli) repulsion and pushes selectivity towards ‘two-conformer behavior’. In contrast, stabilizing the ^3^*H*_4_ conformation with a C5-CO_2_CH_3_ moiety (as in mannuronic acid) widens the energy gap between the ^3^*H*_4_ and *B*_2,5_ conformers, enforcing exclusive β-selectivity.

Together, these findings extend the mechanistic framework for S_N_1-type glycosylations beyond the classical two-conformer model. They show that higher-energy boat-like intermediates can control stereochemistry when they offer a lower-repulsion reaction trajectory and that small, targeted changes in ring substitution can be used to bias glycosylation outcomes in a predictable way. This insight provides a quantitative foundation for designing stereoselective glycosylation reactions and highlights the need to critically (re-)examine other oxocarbenium ion conformers for different glycosyl configurations, as similarly unconventional boat-like or other high-energy intermediates may play underappreciated roles in determining selectivity.

## Author contributions

JDCC, TH, FMB: conceptualisation; DH, WAR, JS: investigation; JDCC, TH, FMB: supervision; DH, WAR, JDCC: writing – original draft; DH, WAR, JS, FMB, TH, JDCC: writing – review & editing.

## Conflicts of interest

There are no conflicts to declare.

## Supplementary Material

SC-OLF-D6SC02312F-s001

## Data Availability

The data supporting this article have been included as part of the supplementary information (SI). Supplementary information is available. See DOI: https://doi.org/10.1039/d6sc02312f.
