# Supplementary material for: Beyond the two-conformer model: boat conformers provide stereoselectivity in SN1-type glycosylations of manno-type donors
Source: Chem Sci. 2026 Apr 17;17(21):10708–19. doi: 10.1039/d6sc02312f (PMC13098399; doi:10.1039/d6sc02312f)
Supplement: SC-017-D6SC02312F-s001 [file SC-017-D6SC02312F-s001.pdf]

Supplementary Information (SI) for Chemical Science.
This journal is © The Royal Society of Chemistry 2026

Supplementary Information

Beyond the Two-Conformer Model: The Role of Boat Conformers in the Stereoselectivity of S_N1-Type Glycosylations

Wouter A. Remmerswaal[1,2], Daan Hoogers[1,2], Joeri Schoenmakers[1], F. Matthias Bickelhaupt[3,4,5], Thomas Hansen[3,*], Jeroen D. C. Codée[1,*]

[1]*Leiden University, Leiden Institute of Chemistry, Einsteinweg 55, 2333 CC Leiden, The Netherlands.*
[2]*These authors contributed equally.*
[3]*Department of Chemistry and Pharmaceutical Sciences, Amsterdam Institute of Molecular and Life Sciences (AIMMS), Vrije Universiteit Amsterdam, De Boelelaan 1108, 1081 HZ Amsterdam, The Netherlands*
[4]*Institute for Molecules and Materials, Radboud University, Heyendaalseweg 135, 6525 AJ Nijmegen, The Netherlands.*
[5]*Department of Chemical Sciences, University of Johannesburg, Auckland Park, Johannesburg 2006, South Africa*
**Corresponding author, email address: jcodee@chem.leidenuniv.nl.*

General Computational methods

Using density functional theory (DFT), the potential energy surfaces (PES) of glycosyl cations were calculated. The DFT computations were performed using Gaussian 09 rev D.01.[1] For all computations, the hybrid functional B3LYP[2–4] and the 6-311G(d,p)[5] basis set were used. The geometry convergence criteria were set to tight (opt=tight; max. force=$1.5\cdot10^{-7}$, max. displacement=$6.0\cdot10^{-7}$), and an internally defined super-fine grid size was used (SCF=tight, int=veryfinegrid), which is a pruned 175,974 grid for first-row atoms and a 250,974 grid for all other atoms. These parameters were chosen as recent literature indicated a significant dependence of the computed frequencies on the molecule orientation when a smaller grid size is used.[6] Geometries were optimized without symmetry constraints. All calculated stationary points have been verified by performing a vibrational analysis, to be energy minima (no imaginary frequencies) or transition states (only one imaginary frequency). The character of the normal mode associated with the imaginary frequency of the transition state has been analyzed to ensure that it is associated with the reaction of interest. If a transition state could not be located, due to instability of the associated reactant complex, a constrained potential energy surface was constructed to estimate the barrier height.[7] Solvation in CH_2Cl_2 was taken into account in the computations using the PCM solvation model. Solvent effects were explicitly used in the solving of the SCF equations and during the optimization of the geometry and the vibrational analysis. The potential energy surfaces of the studied addition reactions were obtained by performing intrinsic reaction coordinate (IRC) calculations, which, in turn, were analyzed using the PyFrag program.[8]

The denoted free Gibbs energy was calculated using Equation S1, in which $\Delta E_{\text{dichloromethane}}$ is the solution-phase energy (electronic energy), $\Delta G_{\text{dichloromethane,QH}}$ (T = 213.15 K, C = 1 M standard state) is the sum of corrections from the electronic energy to the free Gibbs energy in the quasi-harmonic oscillator approximation, including zero-point-vibrational energy. The $\Delta G_{\text{gas,QH}}$ was computed using the quasi-harmonic approximation in the solution phase according to the work of Truhlar. The quasi-harmonic approximation is the same as the harmonic oscillator approximation except for those vibrational frequencies lower than 100 cm^{-1} were raised to 100 cm^{-1} as a way to correct for the breakdown of the harmonic oscillator model for the free energies of low-frequency vibrational modes.[9,10]

$$\Delta G_{\text{dichloromethane}} = \Delta E_{\text{dichloromethane}} + \Delta G_{\text{dichloromethane,QH}} \quad \text{(S1)}$$

Solution-phase Activation Strain

The activation strain model (ASM) analysis[11–14] were performed using Gaussian 09 rev D.01.[1] The activation strain model (ASM) of chemical reactivity, also known as the distortion/interaction model, is a fragment-based approach in which the (solution-phase) reaction profiles can be described with respect to, and understood in terms of the characteristics of, the reactants. It considers the rigidity of the reactants and to which extent they need to deform during the reaction, plus their capability to interact with each other as the reaction proceeds. With the help of this model, we decompose the total energy, *i.e.,* $\Delta E_{\text{solution}}(\zeta)$, into the strain and interaction energy, $\Delta E_{\text{solution-strain}}(\zeta)$ and $\Delta E_{\text{solution-int}}(\zeta)$, respectively, and project these values onto the reaction coordinate ζ [Eq. (S2)].

$$\Delta E_{\text{solution}}(\zeta) = \Delta E_{\text{solution-strain}}\ \zeta) + \Delta E_{\text{solution-int}}(\zeta) \quad \text{(S2)}$$

In this equation, the strain energy, $\Delta E_{\text{solution-strain}}(\zeta)$, is the penalty that needs to be paid to deform the reactants from their equilibrium to the geometry they adopt during the reaction at the point ζ of the reaction coordinate. On the other hand, the interaction energy, $\Delta E_{\text{solution-int}}(\zeta)$, accounts for all the chemical interactions that occur between these two deformed reactants along the reaction coordinate. The total strain energy can, in turn, be further decomposed into the strain energies corresponding to the deformation of the cation, $\Delta E_{\text{solution-strain,cation}}(\zeta)$, as well as from the nucleophile, $\Delta E_{\text{solution-strain,nucleophile}}(\zeta)$ [Eq. S3].

$$\Delta E_{\text{solution-strain}}(\zeta) = \Delta E_{\text{solution-strain,cation}}(\zeta) + \Delta E_{\text{solution-strain,allyltrimethylsilane}}(\zeta) \quad \text{(S3)}$$

In order to further analyze the interaction energy, the solution-phase potential energy surface, *i.e.,* $\Delta E_{solution}(\zeta)$, was further decomposed into the $\Delta E_{solvation}(\zeta)$, which accounts for the interaction between the solute and solvent, and the $\Delta E_{solute}(\zeta)$, which is the reaction system in gas-phase with the solution-phase geometry [Eq. S4].[15]

$$\Delta E_{solution}(\zeta) = \Delta E_{solvation}(\zeta) + \Delta E_{solute}(\zeta) \quad (S4)$$

The solute term, $\Delta E_{solute}(\zeta)$, is subsequently decomposed into the solvent-free strain, $\Delta E_{solute\text{-}strain}(\zeta)$, and interaction energy, $\Delta E_{solute\text{-}int}(\zeta)$, which are referred to as solute strain and solute interaction, respectively, to distinguish between the two solution-phase activation strain schemes [Eq. S5].

$$\Delta E_{solution}(\zeta) = \Delta E_{solvation}(\zeta) + \Delta E_{solute\text{-}strain}(\zeta) + \Delta E_{solute\text{-}int}(\zeta) \quad (S5)$$

For clarity reasons, $\Delta E_{solution}$, $\Delta E_{solution\text{-}strain}$, $\Delta E_{solution\text{-}int}$, ΔE_{solute}, $\Delta E_{solute\text{-}strain}$, and $\Delta E_{solute\text{-}int}$ are denoted as ΔE, ΔE_{strain} and ΔE_{int} in all cases, however, one can easily deduce based on the level of theory if the ΔE_{solute} or $\Delta E_{solution}$ is decomposed.

In the herein presented activation strain and accompanied energy decomposition diagrams, the intrinsic reaction coordinate (IRC) is projected onto the carbon–leaving group (C···Si) stretch. This critical reaction coordinate undergoes a well-defined change during the reaction from the reactant complex via the transition state to the product and is shown to be a valid reaction coordinate for studying bimolecular reactions. The ASM has been used to analyze the factors affecting the reaction paths of cycloaddition reactions, nucleophilic substitution reactions, eliminations reactions as well as epoxide opening reactions.[16–20]

Energy Decomposition Analysis

The energy decomposition analysis (EDA)[21] was performed using the Amsterdam Density Functional (ADF2018.105)[22–24] software package based on the solution-phase structures obtained by Gaussian 09 rev D.01. For all computations, the B3LYP functional was used. The basis set used, denoted TZ2P, is of triple-ζ quality for all atoms and has been improved by two sets of polarization functions.[25] The accuracies of the fit scheme (Zlm fit) and the integration grid (Becke grid) were, for all calculations, set to VERYGOOD.[26,27] Relativistic effects were accounted for by using the zeroth-order regular approximation (ZORA).[28,29] The interaction energy, *i.e.,* $\Delta E_{solute\text{-}int}(\zeta)$, between the deformed reactants can be further analyzed in terms of quantitative Kohn-Sham molecular orbital (KS-MO) theory together with a canonical EDA. The EDA decomposes the $\Delta E_{solute\text{-}int}(\zeta)$ into the following three energy terms [Eq. (S6)]:

$$\Delta E_{solute\text{-}int}(\zeta) = \Delta V_{elstat}(\zeta) + \Delta E_{Pauli}(\zeta) + \Delta E_{oi}(\zeta) \quad (S6)$$

Herein, $\Delta V_{elstat}(\zeta)$ is the classical electrostatic interaction between the unperturbed charge distributions of the (deformed) reactants and is usually attractive. The Pauli repulsion, ΔE_{Pauli} (ζ), includes the destabilizing interaction between the fully occupied orbitals of both fragments due to the Pauli principle. The orbital interaction energy, $\Delta E_{oi}(\zeta)$, accounts for, amongst others, charge transfer between the fragments, such as HOMO–LUMO interactions.

Analyzing the TS-α-$^{O}S_2$ reaction paths

The S_E2' reactions between allyl(chloro)dimethylsilane and the glycosyl cations could potentially proceed through a bottom-face addition to boat-like conformation, for which the associated TS-α-$^{O}S_2$ is not a clearly defined transition state due to instability of the associated reactant complex. The electronic potential energy surface was determined from a relaxed potential energy surface scan. The starting point of each scan was the α-boat-like product complex. From here, the C-1···allyl(chloro)dimethylsilane bond was elongated in 100 steps with a fine step size of 0.025 Å, while allowing the rest of the geometry to optimize. As a representative structure for the transition state geometry, the point on the associated relaxed potential energy surface with a similar C···Si bond stretch as the TS-α-$^{4}C_1$ was selected.

Double consistent geometries

To make the comparison in interaction energy, between trajectories towards transition states with major differences in C_1···allyl(chloro)dimethylsilane bond distance, a series of numerical experiments were performed. To this end, double consistent geometries were generated, whereby a consistent geometry near the transition state for TS-β-$^1C_4$, TS-β-$^1S_5$, TS-β-$^1S_3$, TS-α-$^OS_2$ and TS-α-$^4C_1$ at a C···Si bond distance of 1.913 Å were taken from the IRC. The C_1···allyl(chloro)dimethylsilane bond distance was to 2.413 Å (Supplementary Figure S15). Note that these geometries are not optimized, instead, they are taken from the IRC, and key bond distances are constrained to match a selected reference structure.

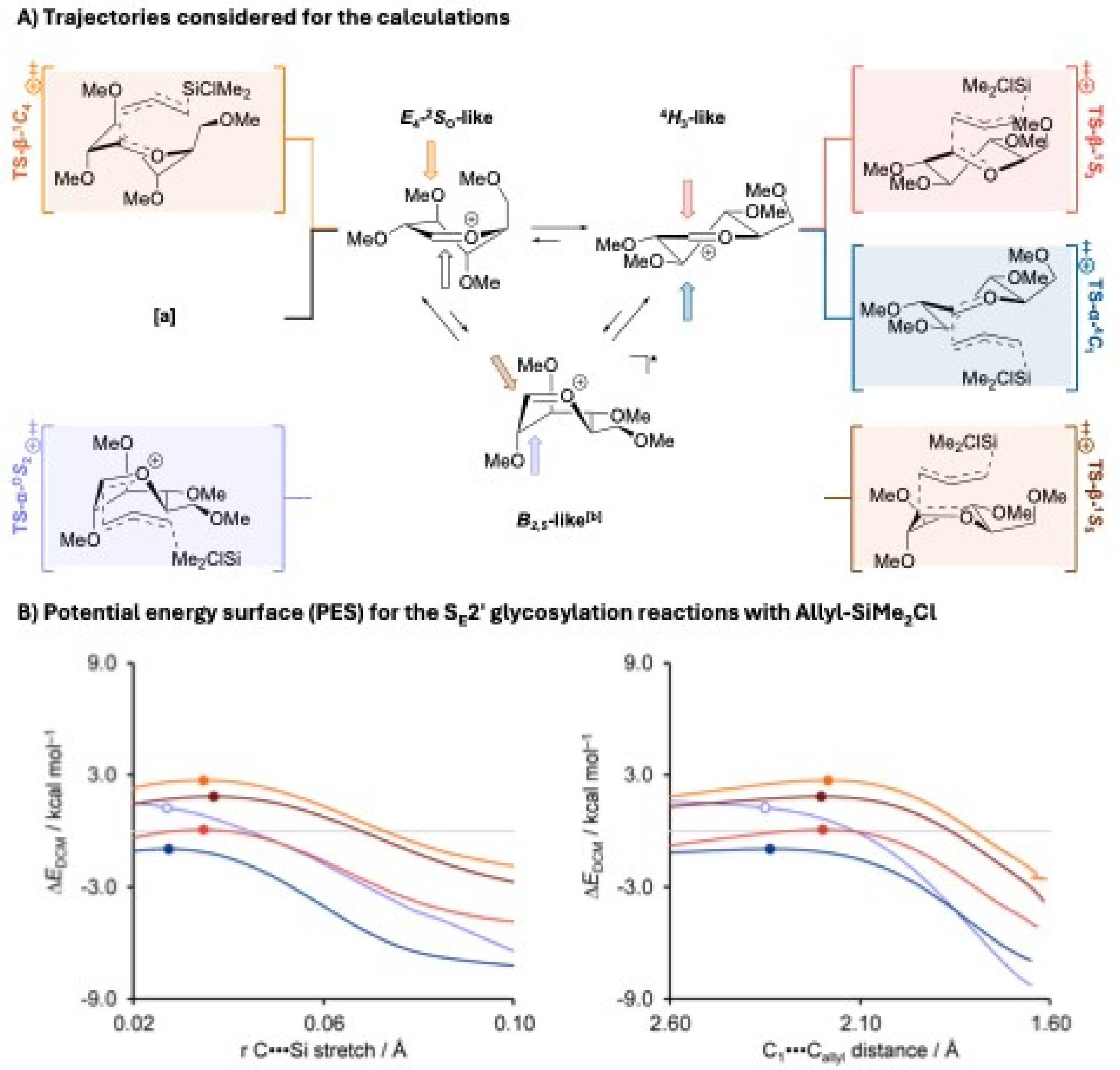

Supplementary Figure S1. (a) The possible S_E2' reaction pathways of the addition of allyl(chloro)dimethylsilane to the glucosyl cation, via the following transition states: TS-β-$^{1}C_4$ (orange), TS-β-$^{1}S_5$ (brown), TS-β-$^{1}S_3$ (red), TS-α-$^{O}S_2$ (light blue) and TS-α-$^{4}C_1$ (blue). (b) Energy values are plotted along the IRC projected on the C···Si bond stretch or C_1···C_{allyl} bond distance. Transition states are indicated by a filled dot. TS-α-$^{O}S_2$ does not exist, instead a TS-like point is indicated by a non-filled dot.[30] Energies are depicted as electronic energies and were computed at PCM(CH_2Cl_2)-B3LYP/6-311G(d,p). [a] A representative structure for the transition state geometry was used.[30] [b] The itinerary leading up to TS-α-$^{O}S_2$ and TS-β-$^{1}S_5$ start from a boat-like non-stationary point.

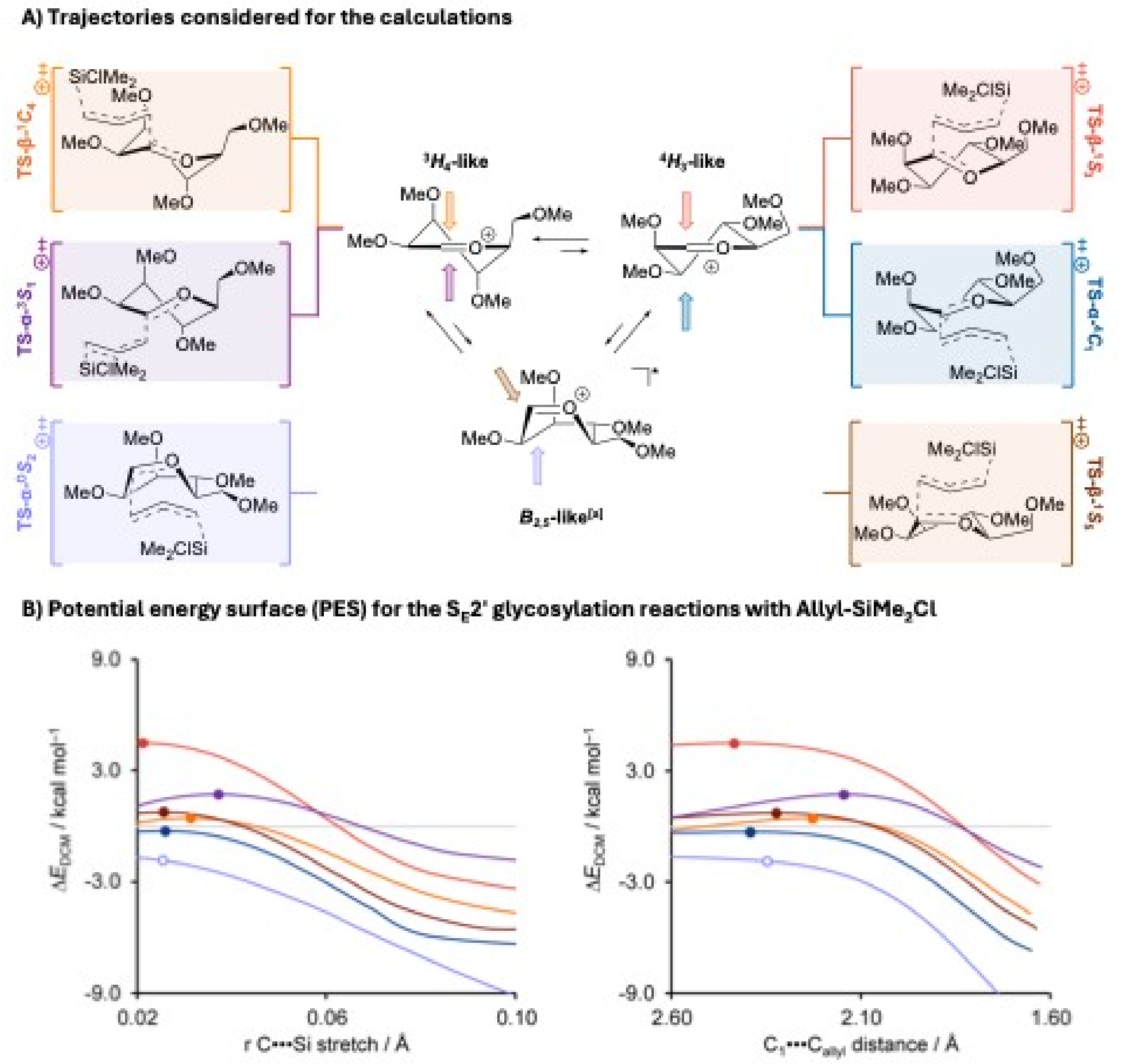

Supplementary Figure S2. (a) The possible S_E2' reaction pathways of the addition of allyl(chloro)dimethylsilane to the mannosyl cation, via the following transition states: TS-β-$^{1}C_{4}$ (orange), TS-β-$^{1}S_{5}$ (brown), TS-β-$^{1}S_{3}$ (red), TS-α-$^{O}S_{2}$ (light blue), TS-α-$^{3}S_{1}$ (purple) and TS-α-$^{4}C_{1}$ (blue). (b) Energy values are plotted along the IRC projected on the C···Si bond stretch or $C_1 \cdots C_{allyl}$ bond distance. Transition states are indicated by a filled dot. TS-α-$^{O}S_{2}$ does not exist, instead a TS-like point is indicated by a non-filled dot.[30] Energies are depicted as electronic energies and were computed at PCM(CH_2Cl_2)-B3LYP/6-311G(d,p). [a] The itinerary leading up to TS-α-$^{O}S_{2}$ and TS-β-$^{1}S_{5}$ start from a boat-like non-stationary point.

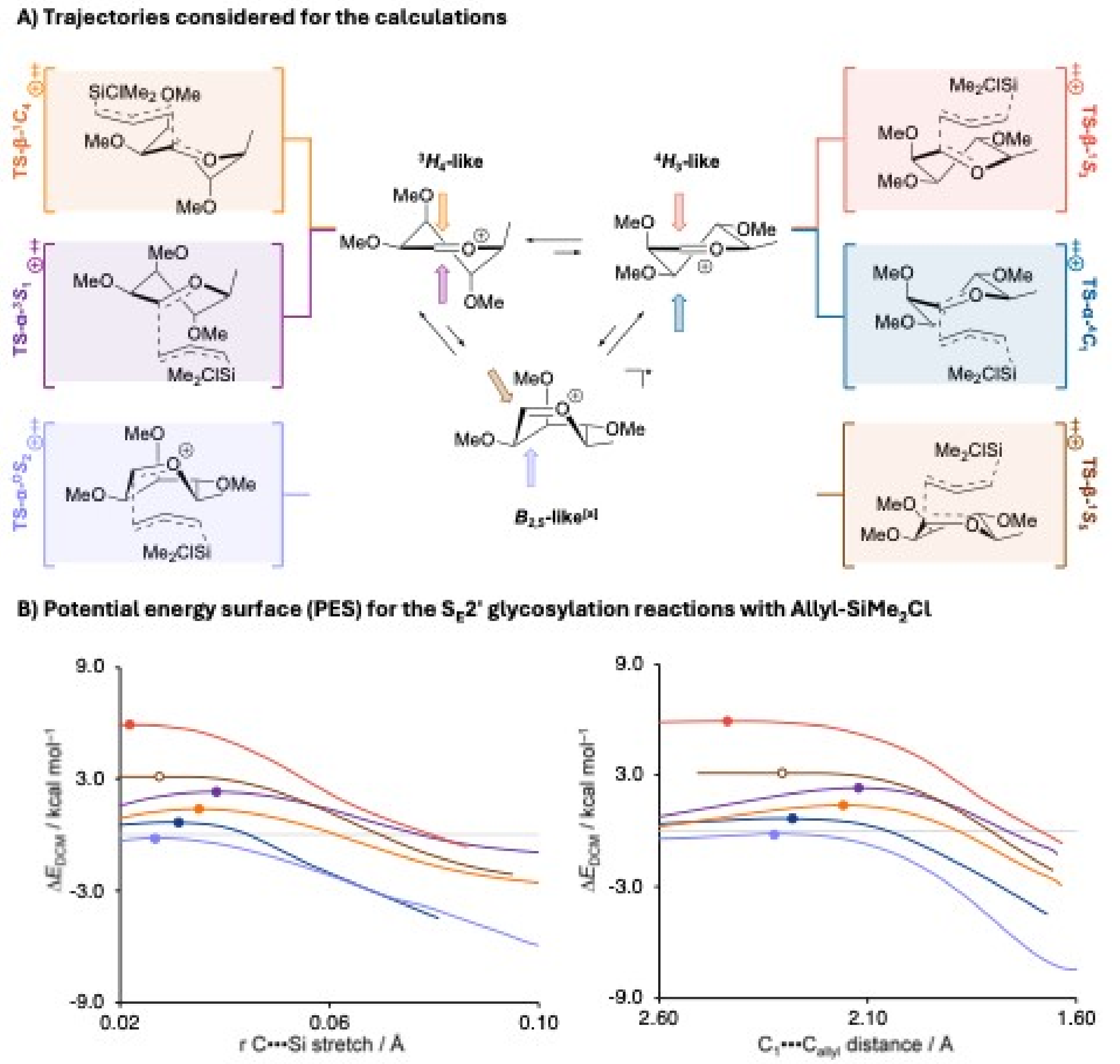

Supplementary Figure S3. (a) The possible S_E2' reaction pathways of the addition of allyl(chloro)dimethylsilane to the rhamnosyl cation, via the following transition states: TS-β-$^{1}C_4$ (orange), TS-β-$^{1}S_5$ (brown), TS-β-$^{1}S_3$ (red), TS-α-$^{O}S_2$ (light blue), TS-α-$^{3}S_1$ (purple) and TS-α-$^{4}C_1$ (blue). (b) Energy values are plotted along the IRC projected on the C···Si bond stretch or C_1···C_{allyl} bond distance. Transition states are indicated by a filled dot. TS-β-$^{1}S_5$ does not exist, instead a TS-like point is indicated by a non-filled dot.[30] Energies are depicted as electronic energies and were computed at PCM(CH_2Cl_2)-B3LYP/6-311G(d,p). [a] The itinerary leading up to TS-α-$^{O}S_2$ and TS-β-$^{1}S_5$ start from a boat-like non-stationary point.

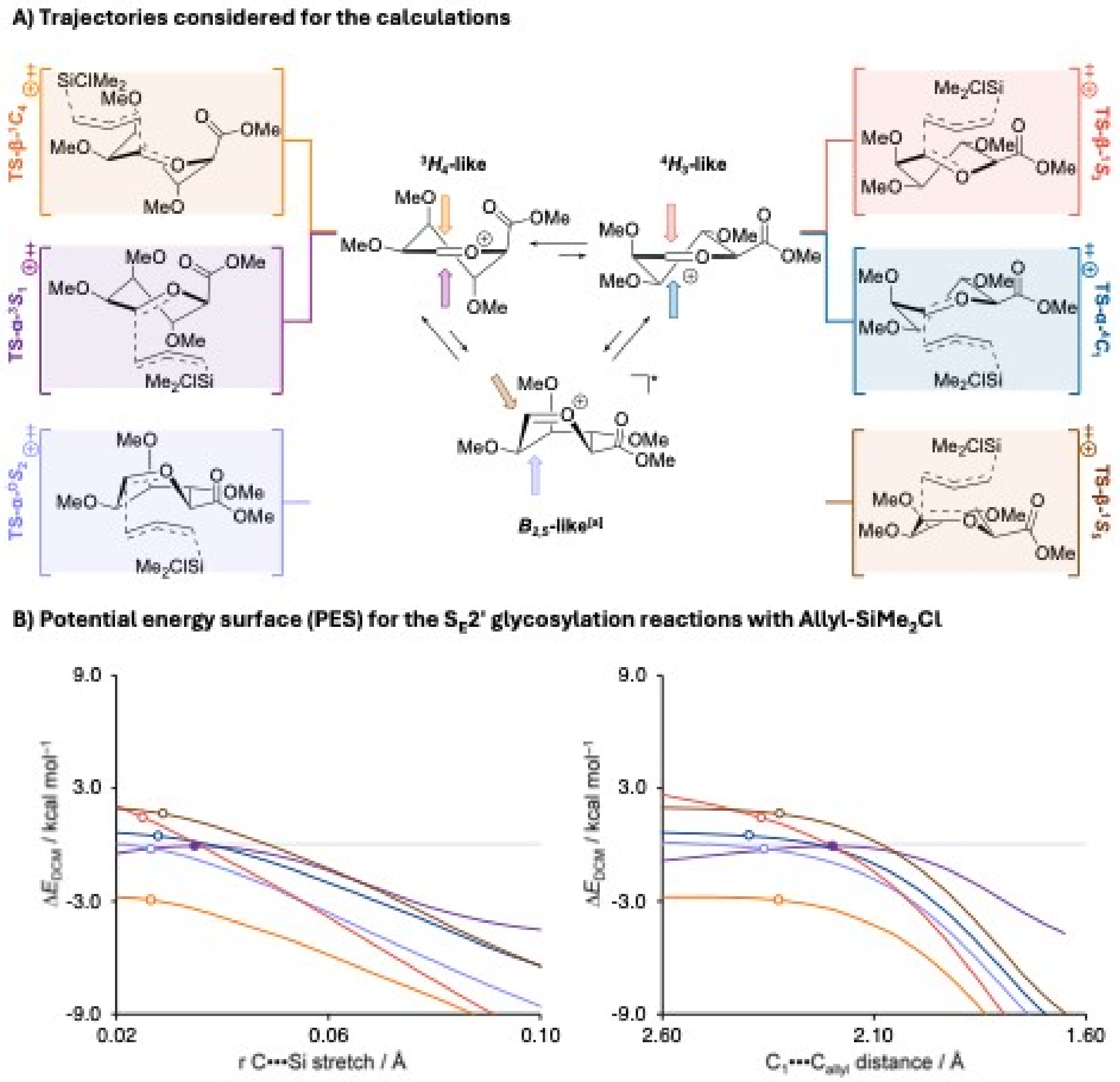

Supplementary Figure S4. (a) The possible S_E2' reaction pathways of the addition of allyl(chloro)dimethylsilane to the mannuronosyl cation, via the following transition states: TS-β-$^1C_4$ (orange), TS-β-$^1S_5$ (brown), TS-β-$^1S_3$ (red), TS-α-$^OS_2$ (light blue), TS-α-$^3S_1$ (purple) and TS-α-$^4C_1$ (blue). (b) Energy values are plotted along the IRC projected on the C···Si bond stretch or $C_1 \cdots C_{allyl}$ bond distance. Transition states are indicated by a filled dot. TS-β-$^1C_4$, TS-β-$^1S_5$, TS-β-$^1S_3$, TS-α-$^OS_2$, and TS-α-$^4C_1$ do not exist, instead a TS-like point is indicated by a non-filled dot.[30] Energies are depicted as electronic energies and were computed at PCM(CH_2Cl_2)-B3LYP/6-311G(d,p). [a] The itinerary leading up to TS-α-$^OS_2$ and TS-β-$^1S_5$ start from a boat-like non-stationary point.

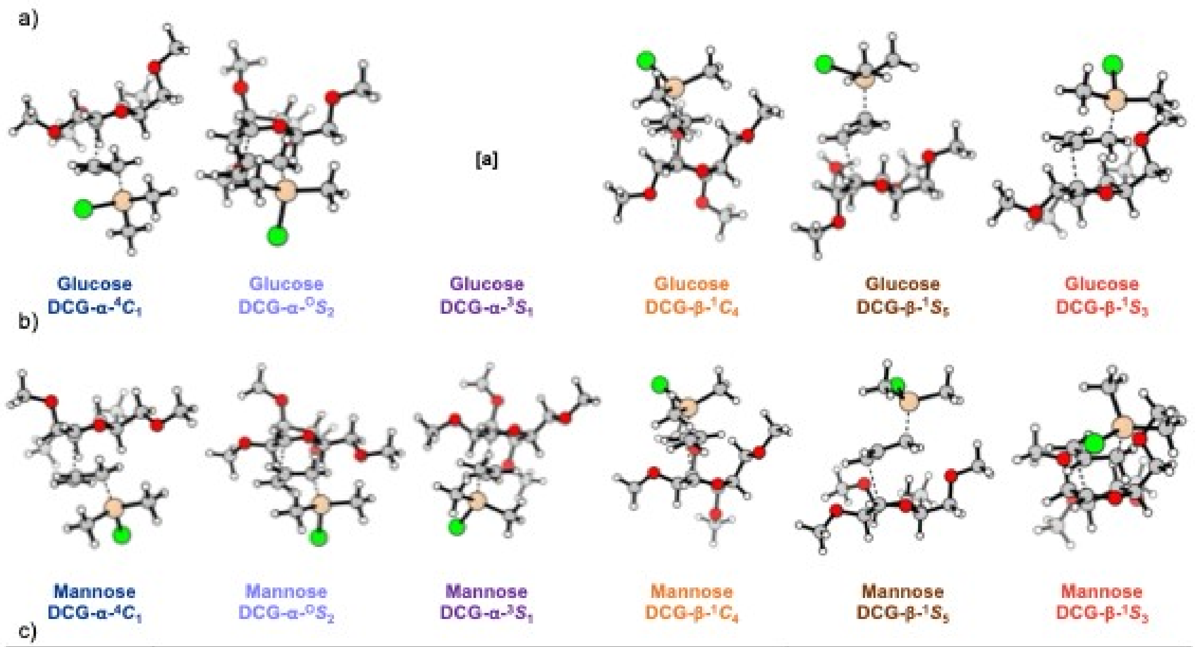

c)

Glucosyl	ΔE_{DCM}	$\Delta E_{DCM\text{-}strain\text{-}total}$	$\Delta E_{DCM\text{-}strain\text{-}cation}$	$\Delta E_{DCM\text{-}strain\text{-}nucleophile}$	$\Delta E_{DCM\text{-}int}$	$\Delta E_{gas\text{-}int}$	$\Delta E_{gas\text{-}Pauli}$	$\Delta E_{gas\text{-}oi}$	$\Delta V_{gas\text{-}elstat}$
CG-α-$^4C_1$	−0.7	+6.9	+5.9	+1.0	−7.6	−11.6	+41.4	−31.1	−21.9
CG-α-$^OS_2$	+1.6	+10.1	+9.0	+1.1	−8.5	−12.0	+37.8	−29.7	−20.1
CG-β-$^1C_4$	+2.4	+9.5	+7.8	+1.7	−7.1	−10.6	+39.9	−28.4	−22.1
CG-β-$^1S_5$	+1.9	+9.2	+7.7	+1.4	−7.3	−11.4	+41.6	−30.1	−22.9
CG-β-$^1S_3$	−0.2	+6.5	+4.8	+1.7	−6.7	−10.3	+40.9	−28.7	−22.5
**Mannosyl**	ΔE_{DCM}	$\Delta E_{DCM\text{-}strain\text{-}total}$	$\Delta E_{DCM\text{-}strain\text{-}cation}$	$\Delta E_{DCM\text{-}strain\text{-}nucleophile}$	$\Delta E_{DCM\text{-}int}$	$\Delta E_{gas\text{-}int}$	$\Delta E_{gas\text{-}Pauli}$	$\Delta E_{gas\text{-}oi}$	$\Delta V_{gas\text{-}elstat}$
CG-α-$^4C_1$	−0.2	+7.0	+5.6	+1.4	−6.8	−11.7	+38.5	−29.3	−20.9
CG-α-$^OS_2$	−1.7	+7.3	+5.8	+1.5	−9.0	−13.1	+35.6	−28.9	−19.8
CG-α-$^3S_1$	1.1	+6.6	+4.9	+1.7	−5.5	−9.6	+36.5	−26.1	−20.0
CG-β-$^1C_4$	+0.2	+8.4	+6.8	+1.6	−8.2	−11.8	+39.6	−29.2	−22.2
CG-β-$^1S_5$	+0.7	+10.7	+9.1	+1.6	−10.0	−13.8	+40.1	−31.6	−22.3
CG-β-$^1S_3$	+4.8	+12.2	+11.3	+0.9	−7.4	−10.1	+41.5	−30.5	−21.1

Supplementary Figure S5. Illustrations[31] of double consistent (C···Si and $C_1 \cdots C_{allyl}$ bond distance) geometries of S_E2' reactions of allyl(chloro)dimethylsilane + (a) glucosyl cations *via* TS-α-$^4C_1$, TS-α-$^OS_2$, TS-α-$^3S_1$, TS-β-$^1C_4$, TS-β-$^1S_5$ and TS-β-$^1S_3$ pathways, and (b) mannosyl cation *via* TS-α-$^4C_1$, TS-α-$^OS_2$, TS-α-$^3S_1$, TS-β-$^1C_4$, TS-β-$^1S_5$ and TS-β-$^1S_3$ pathways. All C···Si and $C_1 \cdots C_{allyl}$ bond distances were set to 1.913 Å and 2.413 Å respectively. (c) Energies at this geometry are given and were computed at PCM(CH_2Cl_2)-B3LYP/6-311G(d,p) (ΔE_{DCM}) and for the EDA analysis at ZORA-B3LYP/TZ2P//PCM(CH_2Cl_2)-B3LYP/6-311G(d,p) (ΔE_{gas}).

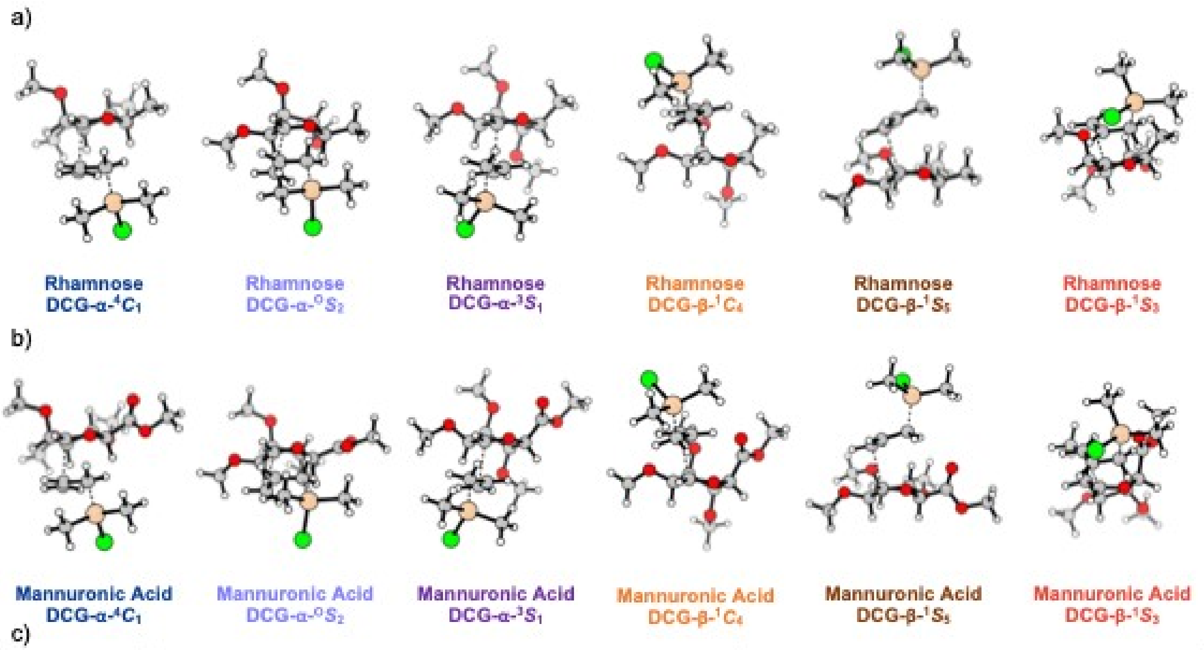

c)

Rhamnosyl	ΔE_{DCM}	$\Delta E_{DCM\text{-}strain\text{-}total}$	$\Delta E_{DCM\text{-}strain\text{-}cation}$	$\Delta E_{DCM\text{-}strain\text{-}nucleophile}$	$\Delta E_{DCM\text{-}int}$	$\Delta E_{gas\text{-}int}$	$\Delta E_{gas\text{-}Pauli}$	$\Delta E_{gas\text{-}oi}$	$\Delta V_{gas\text{-}elstat}$
CG-α-$^4C_1$	+0.9	+7.6	+6.1	+1.5	−6.7	−11.8	+37.8	−29.2	−20.4
CG-α-$^OS_2$	+0.1	+6.5	+5.3	+1.2	−6.4	−11.1	+36.3	−28.0	−19.5
CG-α-$^3S_1$	+1.5	+6.4	+4.7	+1.7	−4.9	−9.0	+37.1	−25.9	−20.2
CG-β-$^1C_4$	+0.9	+7.1	+5.6	+1.5	−6.3	−10.8	+38.4	−27.9	−21.2
CG-β-$^1S_5$	+3.7	+16.4	+13.7	+2.6	−12.7	−17.9	+34.8	−32.6	−17.9
CG-β-$^1S_3$	+5.9	+14.8	+13.6	+1.2	−8.9	−13.2	+36.0	−30.4	−18.8
**Mannuronosyl**	ΔE_{DCM}	$\Delta E_{DCM\text{-}strain\text{-}total}$	$\Delta E_{DCM\text{-}strain\text{-}cation}$	$\Delta E_{DCM\text{-}strain\text{-}nucleophile}$	$\Delta E_{DCM\text{-}int}$	$\Delta E_{gas\text{-}int}$	$\Delta E_{gas\text{-}Pauli}$	$\Delta E_{gas\text{-}oi}$	$\Delta V_{gas\text{-}elstat}$
CG-α-$^4C_1$	+1.3	+8.0	+6.8	+1.2	−6.6	−11.2	+39.6	−29.8	−21.1
CG-α-$^OS_2$	+0.4	+7.8	+6.7	+1.1	−7.5	−11.5	+36.5	−28.5	−19.5
CG-α-$^3S_1$	−0.4	+5.5	+4.0	+1.5	−5.9	−9.7	+37.9	−27.0	−20.6
CG-β-$^1C_4$	−2.6	+6.1	+4.9	+1.2	−8.7	−13.6	+42.2	−32.2	−23.6
CG-β-$^1S_5$	+2.0	+12.1	+10.6	+1.5	−10.1	−15.3	+41.0	−33.2	−23.0
CG-β-$^1S_3$	+1.7	+16.0	+14.5	+1.4	−14.3	−19.3	+41.0	−37.1	−23.2

Supplementary Figure S6. Illustrations[31] of double consistent (C···Si and C_1···C_{allyl} bond distance) geometries of S_E2' reactions of allyl(chloro)dimethylsilane + (a) rhamnosyl cations *via* TS-α-$^4C_1$, TS-α-$^OS_2$, TS-α-$^3S_1$, TS-β-$^1C_4$, TS-β-$^1S_5$ and TS-β-$^1S_3$ pathways, and (b) mannuronosyl cation *via* TS-α-$^4C_1$, TS-α-$^OS_2$, TS-α-$^3S_1$, TS-β-$^1C_4$, TS-β-$^1S_5$ and TS-β-$^1S_3$ pathways. All C···Si and C_1···C_{allyl} bond distances were set to 1.913 Å and 2.413 Å respectively. (c) Energies at this geometry are given and were computed at PCM(CH_2Cl_2)-B3LYP/6-311G(d,p) (ΔE_{DCM}) and for the EDA analysis at ZORA-B3LYP/TZ2P//PCM(CH_2Cl_2)-B3LYP/6-311G(d,p) (ΔE_{gas}).

Supplementary Table S1. PCM(CH_2Cl_2)-B3LYP/6-311G(d,p) Gibbs energies (ΔG_{DCM}, in kcal mol^{-1}) and in parentheses electronic energies (ΔE_{DCM}, in kcal mol^{-1}) in CH_2Cl_2 relative to reactants of the stationary points of the S_E2' reaction between allyl(chloro)dimethylsilane ($CH_2CH=CH_2SiClMe_2$) and the glucosyl or mannosyl oxocarbenium ions. All energies are reported relative to the lowest energy conformer.

Glucosyl Cation + $CH_2CHCH_2SiClMe_2$		
TS-α-$^{O}S_2$	13.5	(1.0)
TS-α-$^{4}C_1$	11.2	(−1.2)
TS-α-$^{3}S_1$	[a]	
TS-β-$^{1}C_4$	15.3	(2.5)
TS-β-$^{1}S_5$	14.3	(1.6)
TS-β-$^{1}S_3$	12.8	(−0.2)
**Mannosyl Cation + $CH_2CHCH_2SiClMe_2$**		
TS-α-$^{O}S_2$	10.8	(−1.9)
TS-α-$^{4}C_1$	12.5	(−0.3)
TS-α-$^{3}S_1$	14.6	(1.7)
TS-β-$^{1}C_4$	13.0	(0.4)
TS-β-$^{1}S_5$	13.4	(0.7)
TS-β-$^{1}S_3$	17.2	(4.5)

Supplementary Table S2. PCM(CH_2Cl_2)-B3LYP/6-311G(d,p) Gibbs energies (ΔG_{DCM}, in kcal mol^{-1}) and in parentheses electronic energies (ΔE_{DCM}, in kcal mol^{-1}) in CH_2Cl_2 relative to reactants of the stationary points of the S_E2' reaction between allyl(chloro)dimethylsilane ($CH_2CH=CH_2SiClMe_2$) and the rhamnosyl or mannuronosyl oxocarbenium ions. All energies are reported relative to the lowest energy conformer.

Rhamnosyl Cation + $CH_2CHCH_2SiClMe_2$		
TS-α-$^{O}S_2$	12.1	(−0.2)
TS-α-$^{4}C_1$	14.3	(0.7)
TS-α-$^{3}S_1$	15.1	(2.3)
TS-β-$^{1}C_4$	14.3	(1.4)
TS-β-$^{1}S_5$	15.9	(3.1)
TS-β-$^{1}S_3$	18.2	(5.9)
Mannuronosyl Cation + $CH_2CHCH_2SiClMe_2$		
TS-α-$^{O}S_2$	12.2	(−0.3)
TS-α-$^{4}C_1$	13.3	(0.5)
TS-α-$^{3}S_1$	12.8	(−0.1)
TS-β-$^{1}C_4$	9.5	(−2.9)
TS-β-$^{1}S_5$	14.4	(1.6)
TS-β-$^{1}S_3$	14.0	(1.4)

Supplementary Table S3. Cartesian coordinates (in Å), energies (*E*, *H* and *G*, in Hartree), and number of imaginary vibrational frequencies (N_{imag}) of all stationary points and transition states, computed at PCM(CH2Cl2)-B3LYP/6-311G(d,p). [a]A representative structure for the transition state geometry was used.[30]

Chlorodimethylallylsilane

***E* =** -947.029573

***H* =** -946.876979

***qh-G* =** -946.906814

N_{imag} = 0

C	3.251175	-0.005117	0.153155
H	3.995963	0.660538	0.574781
H	3.460018	-1.069942	0.202754
C	2.135325	0.465352	-0.402527
H	1.977741	1.541702	-0.429224
C	1.037242	-0.369547	-0.992137
H	0.783863	-0.017242	-1.999304
H	1.357355	-1.414212	-1.088990
Si	-0.571518	-0.399422	0.005144
Cl	-1.277757	1.613985	0.086582
H	-1.235697	-0.901345	2.343421
H	0.440932	-0.326181	2.272867
H	0.051122	-1.982883	1.786456
C	-1.890509	-1.395100	-0.872892
H	-2.063559	-1.020125	-1.884551
H	-2.836313	-1.361649	-0.326350
H	-1.577759	-2.441611	-0.945888
C	-0.304992	-0.947739	1.772754

Glucose-R-$^4H_3$-like

***E* =** -768.544676

***H* =** -768.242619

***qh-G* =** -768.280651

N_{imag} = 0

O	0.766538	-1.325739	-1.380341
C	0.422018	0.896568	-0.165433
C	-1.192468	-1.002837	0.022541
C	-1.058605	0.512890	-0.276614
C	-0.285311	-1.770799	-0.874817
C	1.333594	0.046807	-1.068674
H	0.732326	0.770810	0.876873
H	-0.854286	-1.164678	1.059145
H	-1.415842	0.711510	-1.293862
H	-0.560611	-2.771235	-1.209188
H	1.396127	0.499215	-2.056335
O	-2.503690	-1.451210	-0.187746
O	-1.784950	1.253092	0.667362
O	0.651489	2.220030	-0.602659
C	2.708891	-0.226743	-0.500882
H	3.210573	0.743298	-0.378455
H	3.292469	-0.828252	-1.208648
C	-3.022304	-2.321859	0.831500
H	-2.455838	-3.256715	0.886166
H	-4.049667	-2.538971	0.547824
H	-3.002606	-1.821867	1.804298
C	-3.065240	1.722341	0.221381
H	-3.716263	0.888744	-0.050336

H	-2.952721	2.399158	-0.631801
H	-3.496845	2.266399	1.059853
C	0.730018	3.202711	0.439755
H	0.940730	4.149052	-0.055927
H	1.544895	2.967855	1.132767
H	-0.211118	3.271370	0.987501
O	2.537007	-0.895887	0.727499
C	3.768511	-1.198814	1.380835
H	4.327188	-0.284139	1.610107
H	4.390372	-1.855306	0.761499
H	3.515338	-1.710133	2.308028

Glucose-R-E_4-$^2S_0$-like
***E* =** -768.543155
***H* =** -768.241290
***qh-G* =** -768.278989
N_{imag} = 0

O	-0.173478	-0.954926	-1.836211
C	0.918803	-1.176792	0.336520
C	0.064719	1.087574	-0.527178
C	0.758366	0.321987	0.655167
C	-0.024990	0.288027	-1.774130
C	-0.157940	-1.772965	-0.565103
H	0.904145	-1.729408	1.281991
H	-0.985538	1.216363	-0.216419
H	1.760460	0.761028	0.721328
H	0.043798	0.780623	-2.743689
H	0.200037	-2.734153	-0.925992
O	0.720740	2.301806	-0.782933
O	0.075449	0.452700	1.869112
O	2.127115	-1.378405	-0.379317
C	-1.586257	-1.924375	-0.077878
H	-1.549156	-2.507300	0.853272
H	-2.149491	-2.507298	-0.818472
C	-0.133931	3.376592	-1.199082
H	-0.609513	3.164967	-2.162528
H	0.505014	4.250927	-1.301002
H	-0.907216	3.568289	-0.447990
C	0.143759	1.750729	2.461202
H	-0.430974	2.487807	1.890367
H	1.182565	2.088873	2.542524
H	-0.290207	1.658248	3.455161
C	3.291630	-1.507547	0.441482
H	4.129495	-1.654946	-0.237314
H	3.200312	-2.373331	1.105639
H	3.471444	-0.608587	1.040442
O	-2.175058	-0.659932	0.110940
C	-3.520432	-0.733296	0.582536
H	-3.563461	-1.217736	1.563888
H	-4.151881	-1.284361	-0.123342
H	-3.880356	0.290444	0.668201

Chlorodimethylallylsilane: Glucose-TS-α-$^OS_2$
***E* =** -1715.572728
***H* =** -1715.116028
***qh-G* =** -1715.166109
N_{imag} = 1

C	-0.616194	-0.751206	1.022788
C	-2.954594	-1.031410	0.567188
C	-1.504697	0.741592	-0.602612
C	-2.938896	0.240390	-0.320312
O	-0.657896	0.459594	0.579688
C	-1.590292	-1.775908	0.472588
H	-3.746592	-1.700511	0.213588
H	-1.101396	0.173193	-1.438212
H	-3.484497	1.031289	0.202688
H	-1.598091	-2.649408	1.131588
H	-0.286994	-0.828806	2.048788
O	-3.111494	-0.738210	1.944188
H	-1.928499	2.421992	-1.816512
O	-3.488495	0.011989	-1.606612
O	-1.265492	-2.120007	-0.851912
Si	4.505004	0.360602	0.207388
C	5.520205	-0.398796	1.576188
H	-5.470095	-0.440514	-1.082512
H	-5.159795	0.095386	-2.748612
H	-5.134897	1.281186	-1.417512
C	5.023901	2.092303	-0.254412
H	-4.420195	-0.366213	3.445488
H	-5.079893	-1.393814	2.145988
H	-4.894396	0.366487	1.897388
Cl	4.719806	-0.857897	-1.510712
H	-3.041190	-2.949210	-1.597212
H	-1.980288	-4.075909	-0.694712
H	-1.510389	-3.496508	-2.314012
C	2.630504	0.361999	0.623388
H	2.118403	0.772898	-0.250612
H	2.547303	1.072499	1.454888
C	2.102006	-0.955302	1.005888
H	2.336007	-1.301401	2.010688
C	1.345708	-1.759303	0.213688
H	1.153907	-1.512003	-0.822612
H	1.097509	-2.764803	0.530588
C	-1.400599	2.223992	-0.873012
O	-1.975100	2.937091	0.200688
H	-0.346199	2.497994	-1.011312
C	-1.951503	4.345892	0.005788
H	-2.419803	4.795591	0.880388
H	-0.923003	4.717293	-0.083412
H	-2.512003	4.632591	-0.892812
C	-2.005790	-3.225209	-1.383712
C	-4.456894	-0.520113	2.368288
C	-4.892496	0.252187	-1.704612
H	6.580205	-0.397394	1.310288
H	5.220207	-1.429097	1.779988
H	5.400604	0.183704	2.494788
H	6.073601	2.107405	-0.558212
H	4.908400	2.758203	0.605888
H	4.420901	2.483402	-1.077112

Chlorodimethylallylsilane: Glucose-TS-α-$^{4}C_{1}$
***E* =** -1715.576238
***H* =** -1715.119631
***qh-G* =** -1715.169865

N_{imag} = 1

C	0.502658	-0.359100	-1.060801
C	2.421824	-1.149553	0.407520
C	1.665906	1.276862	0.290340
C	2.860852	0.319098	0.400212
O	0.726801	0.880629	-0.789790
C	1.555289	-1.427913	-0.827690
H	1.849065	-1.347189	1.320838
H	1.080788	1.238978	1.211747
H	2.211692	-1.301736	-1.707757
H	-0.174011	-0.490301	-1.896684
O	3.494379	0.657155	1.618544
Si	-4.465729	0.571628	0.481489
Cl	-5.565708	-0.871212	-0.611691
H	0.634394	-4.411575	-1.860837
C	3.541087	-3.128094	1.181495
H	4.502090	-3.624119	1.050814
C	-5.262644	2.235664	0.205657
H	2.737892	-3.799213	0.868818
H	3.412409	-2.865834	2.237455
C	4.922281	0.750428	1.551428
C	-4.428772	0.029637	2.265675
H	5.257313	1.018170	2.552738
H	5.228968	1.530791	0.846579
H	5.362679	-0.204813	1.258126
C	-2.709167	0.588685	-0.294196
H	-2.158863	1.370175	0.239748
H	-2.863723	0.904640	-1.331723
C	-2.018591	-0.708769	-0.236563
H	-2.363025	-1.472220	-0.931186
C	-0.997780	-1.029637	0.601232
H	-0.640713	-2.048620	0.659408
H	-0.688436	-0.336051	1.376563
O	1.030152	-2.726602	-0.804051
O	3.583629	-1.951708	0.367189
H	3.530671	0.486471	-0.448832
C	2.046553	2.712884	-0.000413
H	2.586949	3.099763	0.873215
H	1.132786	3.308002	-0.129826
O	2.845562	2.746504	-1.162867
C	3.245721	4.064809	-1.519774
H	3.836655	4.529964	-0.721277
H	2.376057	4.698109	-1.734472
H	3.857720	3.979196	-2.416648
C	1.013396	-3.412287	-2.065245
H	2.023832	-3.481279	-2.478675
H	0.353010	-2.919331	-2.785338
H	-5.295796	2.490605	-0.855937
H	-4.696684	3.011896	0.729593
H	-6.284123	2.239242	0.594094
H	-5.439735	0.017627	2.680612
H	-3.829008	0.730693	2.854210
H	-3.999472	-0.968364	2.377091

Chlorodimethylallylsilane: Glucose-TS-β-$^{1}C_{4}$

***E* =** -1715.570328

***H* =** -1715.113240

***qh-G* =** -1715.163215
***N$_{imag}$* =** 1

C	-0.894053	-0.883852	-0.735377
C	-2.855686	-0.716775	0.892853
C	-2.200554	1.182469	-0.682124
C	-3.355297	0.443620	0.007309
O	-1.231367	0.244255	-1.286179
C	-1.868278	-1.640545	0.166276
H	-3.719682	-1.327160	1.170572
H	-2.570792	1.698925	-1.566049
H	-0.300409	-1.493821	-1.406795
O	-4.253487	-0.101712	-0.938870
O	-2.567949	-2.480262	-0.733355
Si	4.391199	-0.077688	-0.360780
C	5.642603	-0.926880	-1.451158
H	5.582799	-0.527906	-2.468030
H	5.471260	-2.004732	-1.491711
H	6.653534	-0.751020	-1.075008
Cl	4.525723	-0.920009	1.573814
H	-5.819847	1.240669	-0.627170
H	-5.864349	0.255719	-2.112983
H	-4.755540	1.638253	-2.005012
C	4.638149	1.764516	-0.206918
H	3.920996	2.219052	0.479757
H	4.525316	2.236552	-1.187546
H	5.645205	1.980757	0.158395
C	2.627182	-0.515593	-1.003030
H	2.586597	-1.606870	-1.057861
H	2.619177	-0.092539	-2.014916
C	1.552801	0.050503	-0.186922
H	1.347721	1.111438	-0.314047
C	0.758523	-0.650182	0.675853
H	0.966163	-1.699397	0.862076
H	0.147862	-0.140669	1.410561
H	-1.332403	-2.221120	0.918274
O	-2.181714	-0.217797	2.039105
H	-3.873766	1.163348	0.653595
C	-1.478948	2.203141	0.187180
H	-1.071003	1.750598	1.096548
H	-2.234257	2.940018	0.495312
O	-0.464629	2.817732	-0.584392
C	0.099326	3.955580	0.061801
H	0.562936	3.683860	1.018078
H	-0.662954	4.722971	0.242138
H	0.860152	4.356087	-0.606880
C	-2.073945	-3.823420	-0.806461
H	-1.041466	-3.859349	-1.168389
H	-2.716351	-4.343548	-1.514396
H	-2.133991	-4.309007	0.172781
C	-3.027426	0.026589	3.166480
H	-3.521469	-0.897175	3.484711
H	-3.785843	0.786136	2.949802
H	-2.381656	0.386903	3.965458
C	-5.218539	0.819677	-1.441705

Chlorodimethylallylsilane: Glucose-TS-β-$^{1}S_{5}$
***E* =** -1715.571760

***H* =** -1715.114798
***qh-G* =** -1715.164851
N_{imag} = 1

C	-0.823637	-1.089966	-1.175996
C	-2.684844	-1.008541	0.579657
C	-2.241027	0.856551	-1.086329
C	-2.470171	0.506897	0.394354
O	-1.090088	0.099210	-1.626436
C	-1.928882	-1.854795	-0.466771
H	-3.750188	-1.215660	0.427413
H	-3.105060	0.547095	-1.678408
H	-0.135481	-1.629327	-1.816010
O	-3.630369	1.190209	0.841126
O	-2.836753	-2.187872	-1.510733
Si	4.231692	0.122445	0.007159
C	4.184310	1.732482	0.946779
H	-2.594796	-4.259282	-1.425187
C	-3.205451	-1.254437	2.917887
H	-4.193789	-1.634013	2.637420
C	5.617653	-0.001205	-1.233904
H	-3.292217	-0.200504	3.194359
H	-2.829579	-1.818646	3.770432
C	-3.397695	2.317949	1.691294
Cl	4.452654	-1.446617	1.405410
H	-2.918180	2.011822	2.627687
H	-4.377066	2.739682	1.912180
H	-2.774829	3.072408	1.204719
C	2.569174	-0.230576	-0.906959
H	2.675686	-1.225606	-1.346654
H	2.551477	0.526552	-1.699976
C	1.397037	-0.112621	-0.041705
H	1.070027	0.898547	0.192850
C	0.657668	-1.149575	0.453374
H	0.986941	-2.171730	0.293497
H	-0.043384	-0.987695	1.263576
H	-1.534738	-2.746777	0.022292
O	-2.267842	-1.457368	1.857598
H	-1.594203	0.814632	0.972883
C	-1.927828	2.309512	-1.367030
H	-2.827102	2.907618	-1.172151
H	-1.674453	2.415600	-2.429538
O	-0.848134	2.716324	-0.547148
C	-0.404853	4.039508	-0.835160
H	-1.209279	4.768885	-0.682860
H	-0.043967	4.118121	-1.867662
H	0.412050	4.258470	-0.148908
C	-2.597228	-3.446743	-2.157953
H	-1.655603	-3.449118	-2.714022
H	-3.420897	-3.585365	-2.855630
H	5.139703	1.902811	1.449470
H	3.395087	1.741439	1.701570
H	4.012659	2.563274	0.255770
H	6.583680	0.112742	-0.735872
H	5.522007	0.794497	-1.978646
H	5.606833	-0.962896	-1.751271

Chlorodimethylallylsilane: Glucose-TS-β-$^{1}S_{3}$

***E* =** -1715.574578
***H* =** -1715.117450
***qh-G* =** -1715.167335
N_{imag} = 1

C	0.960482	1.210697	-0.835581
C	3.199685	0.251461	-0.113164
C	1.471839	-1.144098	-1.273793
C	2.429064	-1.069026	-0.078970
O	0.789840	0.139313	-1.553476
C	2.219703	1.428705	0.024532
O	4.124596	0.274685	0.952253
H	-0.201355	-2.224361	-2.078195
O	3.293772	-2.175942	-0.246063
O	2.848101	2.596968	-0.454801
H	0.598483	2.095302	-1.345005
H	3.716573	0.332406	-1.077428
H	2.066849	-1.315353	-2.172478
H	1.871653	-1.154896	0.859867
H	1.972272	1.522561	1.083683
Si	-4.207884	0.407295	-0.108219
C	-5.048642	2.053588	0.135728
H	2.768704	-3.251799	1.466690
C	5.390427	0.867863	0.646114
H	5.891266	0.321126	-0.160323
C	-5.168526	-0.796203	-1.160859
H	5.989143	0.796520	1.553304
H	5.280081	1.917322	0.361930
C	2.452202	3.811140	0.194906
Cl	-3.929870	-0.466541	1.796786
H	3.073074	4.599678	-0.225719
H	2.622062	3.743057	1.274278
H	1.400169	4.048449	0.006119
C	-2.445453	0.612329	-0.861459
H	-2.006643	-0.387125	-0.896734
H	-2.647201	0.979141	-1.874860
C	-1.609972	1.548927	-0.113166
H	-1.813253	2.608887	-0.253217
C	-0.587568	1.201134	0.725700
H	-0.438156	0.161029	0.994584
H	-0.160987	1.957668	1.371731
C	0.426516	-2.234776	-1.178353
H	0.963760	-3.190706	-1.145883
O	-0.352393	-2.047833	-0.012192
C	-1.216290	-3.150144	0.259181
H	-1.770520	-2.904297	1.163228
H	-1.921167	-3.312188	-0.565154
H	-0.638596	-4.067435	0.420311
C	3.655881	-2.849906	0.964293
H	4.191038	-2.181182	1.640768
H	4.305095	-3.673657	0.670016
H	-5.159643	2.562056	-0.826814
H	-4.481042	2.702483	0.806289
H	-6.045429	1.911358	0.560484
H	-6.144475	-0.995676	-0.711293
H	-4.638620	-1.744940	-1.270200
H	-5.332397	-0.374009	-2.156690

Mannose-R-$^4H_3$-like
***E* =** -768.542528
***H* =** -768.239911
***qh-G* =** -768.277870
***N*$_{imag}$ =** 0

O	0.701228	-1.504948	-1.257093
C	0.129117	0.692143	-0.170023
C	-1.570032	-1.105225	-0.479205
C	-1.325690	0.402400	-0.549283
C	-0.460930	-1.924231	-1.053301
C	1.087178	-0.038597	-1.122884
H	0.305084	0.353893	0.853496
H	-1.497747	0.729721	-1.582945
H	-0.589184	-2.994935	-1.198022
H	0.984153	0.343142	-2.139202
O	-2.222281	1.027738	0.338942
O	0.415409	2.067666	-0.318114
C	2.540087	-0.047431	-0.711736
H	2.914754	0.980362	-0.812551
H	3.107597	-0.683691	-1.403324
C	-2.886539	2.180623	-0.192508
H	-3.525928	2.557677	0.604179
H	-3.506282	1.913649	-1.055601
H	-2.166877	2.950686	-0.480857
C	0.811523	2.731214	0.890197
H	0.005314	2.707022	1.628580
H	1.024265	3.763084	0.615498
H	1.710544	2.273239	1.313562
O	2.632088	-0.513275	0.614522
C	3.975380	-0.592354	1.083868
H	3.930101	-0.954524	2.109679
H	4.458664	0.391797	1.067129
H	4.564118	-1.291102	0.477763
H	-2.532066	-1.378683	-0.916624
O	-1.425167	-1.589325	0.856192
C	-2.554975	-2.314273	1.381614
H	-2.281838	-2.586005	2.398699
H	-2.751890	-3.217970	0.799827
H	-3.433702	-1.665823	1.384701

Mannose-R-$^3H_4$-like
***E* =** -768.545153
***H* =** -768.243322
***qh-G* =** -768.281641
***N*$_{imag}$ =** 0

O	0.588812	-0.181566	-1.456886
C	0.159998	1.109471	0.671583
C	-1.665459	-0.042280	-0.541685
C	-1.012171	0.129408	0.847266
C	-0.630775	-0.443063	-1.534291
C	1.223325	0.563350	-0.288110
H	0.631875	1.266153	1.647458
H	-1.745310	0.569847	1.527565
H	-0.911755	-1.041389	-2.401842
H	1.724557	1.375993	-0.811657
O	-0.497698	-1.083861	1.335328
O	-0.399804	2.307842	0.169826

C	2.265349	-0.380347	0.280843
H	1.804089	-1.325172	0.586874
H	2.679305	0.102048	1.178366
C	-1.373433	-1.831291	2.193262
H	-0.793802	-2.681041	2.549476
H	-2.250051	-2.181880	1.646842
H	-1.684517	-1.220376	3.046843
C	0.323184	3.498446	0.498817
H	-0.242861	4.327075	0.078027
H	1.328568	3.496608	0.064540
H	0.395894	3.616704	1.584724
O	3.255891	-0.568214	-0.703447
C	4.292505	-1.447094	-0.277597
H	4.997356	-1.527279	-1.103764
H	3.896606	-2.442078	-0.040787
H	4.811637	-1.050543	0.603791
H	-2.010746	0.957396	-0.854732
O	-2.686194	-1.002060	-0.543597
C	-3.917689	-0.580976	-1.152371
H	-4.619724	-1.399840	-1.011685
H	-3.789164	-0.393757	-2.222752
H	-4.299024	0.320655	-0.663375

Chlorodimethylallylsilane: Mannose-TS-α-$^{O}S_{2}$[30]
***E* =** -1715.577684
***H* =** -1715.121185
***qh-G* =** -1715.171250
N_{imag} = 1

C	1.019500	-0.896400	-0.742796
C	-0.785910	-1.696590	0.529204
C	3.280400	-0.324200	-0.114196
C	1.311600	1.308200	0.040104
C	2.832900	1.118900	0.215604
O	0.783600	0.352000	-0.950896
C	2.100400	-1.291600	0.225504
H	4.134400	-0.583310	0.520404
H	0.825100	1.074700	0.989204
H	3.378210	1.785800	-0.461396
O	2.357890	-2.655000	0.033704
H	0.798000	-1.547800	-1.577596
O	3.622200	-0.368700	-1.482496
H	1.178910	2.834100	-1.463596
O	3.067000	1.453300	1.568904
H	1.811900	-1.075700	1.262504
Si	-4.211400	-0.389480	-0.217696
Cl	-4.071300	-0.389380	1.896204
H	3.062890	-4.356600	0.879404
H	2.349890	-3.212800	2.046404
H	3.969190	-2.902310	1.356204
C	-5.120110	-1.938580	-0.724996
H	-5.174710	-1.995780	-1.816496
H	-6.140710	-1.930280	-0.334596
H	-4.619610	-2.837480	-0.358196
C	-5.058300	1.192120	-0.731296
H	-5.135900	1.236120	-1.821696
H	-4.505090	2.069820	-0.389596
H	-6.068400	1.236520	-0.316296

C	-2.405800	-0.371890	-0.865796
H	-1.945600	0.540410	-0.474096
H	-2.511900	-0.273990	-1.952296
C	-1.636910	-1.578690	-0.522296
H	-1.757410	-2.439590	-1.176396
H	-0.716400	-0.910690	1.273804
H	-0.333510	-2.651990	0.764204
C	0.897810	2.685900	-0.411296
O	-0.495890	2.810710	-0.226596
H	1.446410	3.416300	0.199104
C	-0.997790	4.079610	-0.633796
H	-2.069690	4.073910	-0.440596
H	-0.532290	4.890610	-0.060696
H	-0.822990	4.251210	-1.702796
C	2.973590	-3.306900	1.151404
C	4.577800	-1.370810	-1.852096
C	4.384710	1.937790	1.841104
H	5.148400	1.182890	1.626904
H	4.409810	2.177390	2.902704
H	4.594410	2.839890	1.256604
H	4.836900	-1.175210	-2.891596
H	4.160290	-2.375210	-1.759396
H	5.479500	-1.288410	-1.234896

Chlorodimethylallylsilane: Mannose-TS-α-$^{4}C_{1}$

***E* =** -1715.575170

***H* =** -1715.118256

***qh-G* =** -1715.168565

N_{imag} = 1

C	0.775070	-0.829872	-1.151502
C	-0.792427	-1.619560	0.475376
C	2.611840	-0.766341	0.604354
C	1.292336	1.264119	-0.055161
C	2.689973	0.732788	0.296829
O	0.632611	0.445414	-1.105052
C	2.010926	-1.511455	-0.599710
H	1.953770	-0.897082	1.473523
H	0.645180	1.187557	0.821293
O	2.885339	-1.384435	-1.713239
H	0.292597	-1.283278	-2.009124
O	3.084629	1.463608	1.439945
Si	-4.321256	-0.368084	-0.008916
C	-5.198682	1.209012	-0.484332
H	-5.345483	1.240867	-1.568221
H	-4.625246	2.090358	-0.188260
H	-6.180344	1.259235	-0.006719
Cl	-4.022671	-0.338981	2.087614
H	4.609388	2.386006	2.404585
H	4.729055	2.469664	0.629394
H	5.107377	0.942874	1.480685
C	-5.272383	-1.918793	-0.425957
H	-4.753507	-2.817974	-0.086549
H	-5.411634	-1.988368	-1.509178
H	-6.260037	-1.896796	0.041447
C	-2.570472	-0.363433	-0.794255
H	-2.091385	0.566060	-0.474984
H	-2.763860	-0.305502	-1.871824

C	-1.758991	-1.551145	-0.474373
H	-1.960781	-2.448470	-1.055391
H	-0.335688	-2.569715	0.722165
H	-0.626597	-0.796668	1.162064
H	1.807218	-2.557625	-0.368626
O	3.906172	-1.241924	0.890633
H	3.379766	0.893956	-0.539138
C	1.264996	2.686142	-0.566832
H	1.676773	2.725097	-1.585050
H	1.900830	3.291073	0.090780
O	-0.074372	3.133195	-0.537263
C	-0.224224	4.459984	-1.029043
H	0.086595	4.532393	-2.078747
H	0.361397	5.172055	-0.434468
H	-1.281152	4.710865	-0.949681
C	3.197148	-2.607205	-2.396930
H	3.683408	-3.309816	-1.714940
H	2.302276	-3.065664	-2.827556
H	3.882988	-2.336240	-3.197139
C	3.951354	-2.316724	1.830904
H	5.003267	-2.560738	1.970253
H	3.428253	-3.205426	1.461047
H	3.517688	-2.015585	2.790758
C	4.469513	1.828460	1.479434

Chlorodimethylallylsilane: Mannose-TS-α-$^{3}S_{1}$
***E* =** -1715.571973
***H* =** -1715.114969
***qh-G* =** -1715.165257
N_{imag} = 1

Cl	-4.774421	-0.845661	1.282842
H	5.019175	-1.668276	-2.194490
H	4.038438	2.268944	0.484823
H	5.120948	-2.480846	-0.613263
C	-0.987119	-0.368826	0.656253
H	-1.279240	-1.412920	0.655717
H	-0.391041	-0.032887	1.497147
C	-1.709929	0.524998	-0.084976
H	-1.460619	1.580033	0.006871
C	-2.774341	0.182138	-1.025996
H	-2.751743	-0.871337	-1.317518
H	-2.734834	0.816081	-1.919219
Si	-4.546675	0.500296	-0.331107
C	-5.784243	0.040879	-1.647601
H	-5.637252	-0.984152	-1.994656
H	-5.686218	0.714428	-2.504082
H	-6.801123	0.131269	-1.257561
C	-4.729386	2.235336	0.326808
H	-5.744647	2.394275	0.698656
H	3.729409	1.494754	-1.088049
H	-4.031393	2.437664	1.142105
O	1.301600	0.700170	-0.625636
C	2.845769	-0.087876	1.184095
C	1.427541	-1.710392	-0.053545
C	2.883623	-1.334104	0.292016
C	0.725790	-0.467336	-0.632350
C	2.249456	1.112536	0.427753

H	0.184286	-0.638538	-1.556850
H	1.648317	1.713436	1.113739
C	3.258755	2.020345	-0.249586
O	2.581521	3.184285	-0.676825
C	3.433155	4.091388	-1.364512
H	2.822341	4.946455	-1.652198
H	3.859206	3.632626	-2.265680
H	4.253253	4.435193	-0.720900
H	0.953707	-2.049327	0.868844
O	1.420186	-2.710982	-1.040635
C	0.315469	-3.618419	-0.978421
H	0.247453	-4.078653	0.012968
H	0.505428	-4.386340	-1.725826
H	-0.631822	-3.121310	-1.213881
O	2.057615	-0.423598	2.318132
H	3.869137	0.156087	1.486679
C	2.365418	0.323360	3.496458
H	2.167438	1.393146	3.365280
H	3.414052	0.183212	3.779883
H	1.721723	-0.061759	4.285426
O	3.649441	-1.018772	-0.849270
H	3.324072	-2.162961	0.853310
C	4.437800	-2.091509	-1.376426
H	3.805436	-2.897721	-1.751635
H	-4.544715	2.957578	-0.474392

Chlorodimethylallylsilane: Mannose-TS-β-$^{1}C_{4}$

***E* =** -1715.574027

***H* =** -1715.117335

***qh-G* =** -1715.167686

N_{imag} = 1

C	-0.947301	-0.417686	-1.132772
C	-2.888889	-0.706846	0.489523
C	-2.271439	1.549833	-0.542893
C	-3.405733	0.629828	-0.090833
O	-1.244561	0.829607	-1.331052
C	-1.983155	-1.360822	-0.568844
H	-3.750034	-1.360289	0.662311
H	-2.664871	2.246201	-1.281079
H	-0.282152	-0.819330	-1.888107
O	-4.218355	0.378239	-1.227349
H	-2.623663	-1.513396	-1.457394
Si	4.353484	-0.009670	-0.368331
C	5.604946	-0.529070	-1.650229
H	5.600530	0.178537	-2.484544
H	5.385805	-1.525244	-2.040734
H	6.608699	-0.540196	-1.218218
Cl	4.391381	-1.435980	1.193761
H	-5.776012	-0.682928	-0.296043
H	-6.099837	0.024693	-1.894344
H	-6.026777	1.077855	-0.458527
C	4.678443	1.668978	0.378337
H	3.965327	1.907311	1.170449
H	4.606871	2.440115	-0.394798
H	5.685139	1.706355	0.801997
C	2.596322	-0.125574	-1.144027
H	2.496124	-1.151286	-1.509908

H	2.649220	0.565559	-1.994140
C	1.518816	0.251372	-0.224228
H	1.375568	1.316045	-0.052204
C	0.665618	-0.615834	0.391590
H	0.797638	-1.685751	0.286380
H	0.024446	-0.281550	1.195983
O	-1.380337	-2.567275	-0.182777
O	-2.224320	-0.442219	1.708339
H	-3.971386	1.149392	0.690191
C	-1.598909	2.338505	0.573388
H	-1.205324	1.680797	1.354072
H	-2.378251	2.966259	1.028636
O	-0.579112	3.135809	0.004821
C	-0.035421	4.066705	0.935572
H	0.408873	3.555685	1.798855
H	-0.804463	4.762629	1.292256
H	0.738109	4.625854	0.410643
C	-2.137682	-3.741391	-0.501701
H	-1.535540	-4.588367	-0.178748
H	-2.313011	-3.808335	-1.580173
H	-3.095934	-3.760907	0.026375
C	-2.365989	-1.451237	2.715894
H	-1.882072	-2.382467	2.413742
H	-3.424316	-1.635003	2.929510
H	-1.882666	-1.061602	3.610613
C	-5.605428	0.187756	-0.938147

Chlorodimethylallylsilane: Mannose-TS-β-$^1S_5$

***E* =** -1715.573538

***H* =** -1715.117093

***qh-G* =** -1715.167123

N_{imag} = 1

C	-1.240107	-0.890783	-1.536043
C	0.243549	-1.629769	0.093116
C	-2.777103	-0.235464	0.414538
C	-1.721739	1.442823	-1.238491
C	-2.081766	1.132112	0.218359
O	-0.950883	0.329618	-1.844994
C	-2.525757	-1.176933	-0.804541
O	-2.308622	-0.775367	1.630452
H	-0.573045	2.766870	-2.466460
O	-2.903717	2.208778	0.622913
H	-3.280624	-0.893049	-1.562077
H	-0.840458	-1.625958	-2.221415
H	-3.856537	-0.060536	0.469452
H	-2.626124	1.537390	-1.845313
H	-1.156497	1.120127	0.802174
O	-2.661259	-2.525049	-0.460973
Si	3.840061	-0.483685	0.024598
C	-2.861319	2.498378	2.023303
H	-2.787231	-3.360096	-2.386293
H	-4.349903	-2.981979	-1.604585
H	-3.360280	-4.344870	-1.020851
H	-3.532774	-2.460757	1.832865
H	-4.157651	-0.960200	2.579817
H	-2.772928	-1.810697	3.308861
C	-3.258970	-1.551540	2.369203

H	-3.284975	1.683969	2.617861
C	1.037370	3.806441	-0.612216
H	1.858895	3.666336	0.088899
H	0.457399	4.689135	-0.317680
C	2.168566	-0.356964	-0.908918
H	1.679418	0.560993	-0.569010
H	2.467180	-0.233537	-1.957042
C	1.304387	-1.535479	-0.751290
H	1.531295	-2.393611	-1.381306
H	0.036015	-0.855270	0.819781
H	-0.277916	-2.569192	0.224367
C	-0.881897	2.689756	-1.415609
H	-1.515793	3.551947	-1.177296
O	0.238324	2.629046	-0.557185
H	1.442133	3.965066	-1.619145
H	-3.457714	3.398069	2.167711
H	-1.832721	2.685333	2.349781
C	-3.329389	-3.339470	-1.435600
C	4.826058	1.073858	-0.267773
Cl	3.361185	-0.564010	2.085643
C	4.763605	-2.047708	-0.406902
H	4.985705	-2.063475	-1.478467
H	4.185538	-2.941491	-0.161913
H	5.711052	-2.093867	0.135870
H	5.755577	1.048074	0.306449
H	4.265022	1.964384	0.023875
H	5.083284	1.158903	-1.327733

Chlorodimethylallylsilane: Mannose-TS-β-$^{1}S_{3}$

***E* =** -1715.567572

***H* =** -1715.110937

***qh-G* =** -1715.161104

N_{imag} = 1

C	-0.811181	-1.114779	-0.956985
C	-3.213729	-0.651157	-0.650405
C	-1.579609	1.216252	-1.081896
C	-2.851499	0.800749	-0.326986
O	-0.685625	0.070434	-1.426239
C	-2.039611	-1.541561	-0.153596
O	-4.407922	-0.986842	0.015752
H	0.092698	2.567985	-1.056503
O	-3.835123	1.714489	-0.775178
H	-2.211391	-2.593262	-0.403212
H	-0.269277	-1.853442	-1.532080
H	-3.337595	-0.760999	-1.736415
H	-1.878037	1.566435	-2.070162
H	-2.694920	0.889149	0.750523
O	-1.862422	-1.381985	1.236778
Si	4.333736	0.188175	0.016579
Cl	5.432309	-1.452897	-0.760737
H	-2.074912	-3.391857	1.773103
H	-3.550248	-2.390999	1.950610
H	-2.180425	-2.175706	3.070257
C	5.061981	1.747818	-0.705999
H	4.512950	2.620936	-0.340789
H	6.107672	1.855778	-0.407401
H	5.013593	1.741823	-1.797244

C	4.432923	0.100810	1.877887
H	3.858251	0.920825	2.319333
H	4.032238	-0.841316	2.258122
H	5.469132	0.195339	2.211656
C	2.540903	-0.034885	-0.615995
H	1.984161	0.838872	-0.263398
H	2.616092	0.003997	-1.709024
C	1.902736	-1.289687	-0.174864
H	2.265592	-2.204751	-0.638743
C	0.911924	-1.383221	0.744549
H	0.549272	-0.511103	1.274014
H	0.573655	-2.352291	1.087531
C	-0.751351	2.290650	-0.412111
H	-1.403588	3.168662	-0.309621
O	-0.305784	1.828306	0.845137
C	0.267249	2.858865	1.646827
H	0.568676	2.398632	2.586554
H	1.145310	3.299198	1.159625
H	-0.463863	3.650031	1.848379
C	-4.754782	2.152675	0.231085
H	-5.337141	1.317224	0.623745
H	-5.417536	2.867668	-0.255054
H	-4.226448	2.651426	1.051581
C	-5.246173	-1.906378	-0.689208
H	-5.544824	-1.494467	-1.658757
H	-6.130190	-2.054277	-0.071081
H	-4.752689	-2.872255	-0.842336
C	-2.463574	-2.404096	2.043902

Rhamnose-R-$^4H_3$-like
***E* =** -653.997446
***H* =** -653.729522
***qh-G* =** -653.764383
N_{imag} = 0

C	-0.708212	-1.920004	-0.733773
H	-3.501702	0.116716	0.981071
C	-0.344657	0.583114	-0.593232
C	1.573520	-1.062807	-0.467207
C	0.963701	0.221299	0.113991
O	0.518904	-2.151386	-0.656149
C	-1.329838	-0.587289	-0.478086
H	-0.126596	0.752781	-1.656541
H	1.904322	-0.881715	-1.490344
O	-1.660800	-0.849288	0.887146
H	-1.329253	-2.803385	-0.870783
O	1.953121	1.203002	-0.109444
H	-3.593756	-1.641926	0.647844
H	-2.217173	-0.441912	-1.097188
H	-3.127512	-1.066337	2.268347
C	-1.605165	2.588755	-0.893521
H	-1.952113	3.434445	-0.302512
O	-0.881171	1.739581	0.002138
H	2.938726	2.804057	0.645640
H	2.477383	1.601343	1.875728
H	1.239019	2.687173	1.175933
H	0.765340	0.088924	1.183286
H	-2.471976	2.077230	-1.325454

H	-0.956331	2.947794	-1.699094
C	2.151779	2.124970	0.970424
C	2.653537	-1.700451	0.370951
H	2.277788	-1.963229	1.361501
H	3.462018	-0.976394	0.481510
H	3.050098	-2.590548	-0.117562
C	-3.067786	-0.860967	1.201968

Rhamnose-R-$^3H_4$-like
***E* =** -654.001731
***H* =** -653.73442
***qh-G* =** -653.769314
N_{imag} = 0

C	-0.362387	-0.736850	-1.633507
C	-0.249477	0.129067	0.684102
C	1.861464	-0.527383	-0.601482
C	1.227665	0.460472	0.380895
O	0.852830	-1.021120	-1.645586
C	-1.007462	0.180681	-0.664274
H	-0.663919	0.891786	1.350087
H	2.560595	0.013056	-1.237054
H	-0.960543	-1.218733	-2.407413
O	1.326394	1.753321	-0.191810
H	-0.830945	1.188223	-1.085876
H	-4.267088	0.554717	-0.565364
H	-3.152111	1.541479	-1.544346
H	-1.369190	-0.621033	2.979971
H	-1.217181	-2.357747	2.614882
C	1.515096	2.816721	0.749587
O	-2.366109	-0.149544	-0.598344
H	0.670717	2.906127	1.440213
H	1.595540	3.731201	0.164899
H	2.437550	2.666040	1.319538
O	-0.304271	-1.150077	1.265060
H	-3.098555	1.626524	0.243315
C	-1.384349	-1.372352	2.183444
H	-2.346999	-1.351419	1.670042
H	1.795598	0.395283	1.314383
C	2.472726	-1.785193	-0.023939
H	1.756842	-2.321512	0.596193
H	3.327115	-1.494647	0.590948
H	2.834259	-2.430680	-0.824834
C	-3.263681	0.971619	-0.617182

Chlorodimethylallylsilane: Rhamnose-TS-α-$^OS_2$
***E* =** -1601.031589
***H* =** -1600.609564
***qh-G* =** -1600.656852
N_{imag} = 1

C	0.900748	-0.608476	-0.622127
C	-0.896333	-1.028017	0.799581
C	3.234419	-0.206227	-0.159453
C	1.460540	1.659069	-0.203774
C	2.955262	1.306198	-0.016895
O	0.781901	0.614139	-1.007346
C	1.980323	-0.978458	0.357886
H	4.091067	-0.470881	0.469257

H	0.993843	1.631245	0.782974
H	3.555298	1.816798	-0.777377
O	2.079817	-2.376392	0.360707
H	0.585176	-1.345728	-1.348072
O	3.494179	-0.472106	-1.521676
H	1.597686	2.964269	-1.911265
O	3.259786	1.779340	1.281883
H	1.769018	-0.586912	1.361064
Si	-4.363047	0.028698	-0.308764
Cl	-4.348505	0.477339	1.761550
H	2.626194	-4.008199	1.431833
H	2.152798	-2.611971	2.434455
H	3.741841	-2.635793	1.616543
C	-5.198444	-1.625738	-0.525864
H	-5.174738	-1.919945	-1.579564
H	-6.244300	-1.567968	-0.214173
H	-4.709605	-2.406713	0.060751
C	-5.218985	1.433011	-1.190198
H	-5.237434	1.242256	-2.267301
H	-4.710716	2.384123	-1.016968
H	-6.251401	1.525631	-0.843898
C	-2.518323	-0.019619	-0.840578
H	-2.095302	0.955900	-0.584994
H	-2.555753	-0.116893	-1.932028
C	-1.756968	-1.128960	-0.245244
H	-1.893488	-2.109523	-0.696439
H	-0.801406	-0.098674	1.351558
H	-0.468053	-1.919753	1.239441
C	1.201272	2.975999	-0.894192
H	0.133496	3.196601	-0.924829
H	1.701762	3.767046	-0.332378
C	2.690622	-2.926917	1.534127
C	4.326318	-1.608549	-1.783405
C	4.626784	2.148383	1.476776
H	5.301314	1.292765	1.365028
H	4.700267	2.526300	2.495133
H	4.921426	2.933955	0.772821
H	4.535767	-1.593260	-2.852029
H	3.824202	-2.541826	-1.520587
H	5.269402	-1.529435	-1.231006

Chlorodimethylallylsilane: Rhamnose-TS-α-$^{4}C_{1}$

***E* =** -1601.030199

***H* =** -1600.607817

***qh-G* =** -1600.655217

N_{imag} = 1

C	-0.727787	0.881675	-0.927940
C	0.788168	0.926390	0.774832
C	-2.571014	0.192107	0.677958
C	-1.353428	-1.462709	-0.786024
C	-2.714379	-1.053577	-0.199810
O	-0.656384	-0.310680	-1.420848
C	-1.953343	1.333139	-0.148986
H	-1.899523	-0.055499	1.510682
H	-0.700773	-1.775764	0.032940
O	-2.842707	1.679093	-1.200134
H	-0.254824	1.622886	-1.560576

O	-3.132022	-2.168015	0.564228
Si	4.356338	0.004112	-0.083041
C	5.301073	-1.210146	-1.137472
H	5.477658	-0.784770	-2.129822
H	4.755911	-2.149074	-1.255924
H	6.271115	-1.429271	-0.684324
Cl	4.041863	-0.879036	1.813344
H	-4.685672	-3.352501	1.102659
H	-4.822218	-2.695178	-0.546979
H	-5.131023	-1.641219	0.864303
C	5.229499	1.630956	0.178616
H	4.675291	2.283698	0.856653
H	5.348868	2.146973	-0.778978
H	6.224021	1.465311	0.600011
C	2.600743	0.246901	-0.834686
H	2.141570	-0.744902	-0.861266
H	2.796271	0.583990	-1.859144
C	1.782760	1.223409	-0.106848
H	1.991402	2.274073	-0.297625
H	0.353099	1.709459	1.382922
H	0.629314	-0.096207	1.098526
H	-1.717788	2.198566	0.471916
O	-3.841856	0.554448	1.167381
H	-3.419274	-0.841567	-1.010946
C	-1.430460	-2.531192	-1.853398
H	-2.031027	-2.189934	-2.699014
H	-1.894955	-3.419496	-1.425096
H	-0.432648	-2.794662	-2.206285
C	-3.050205	3.084468	-1.395372
H	-3.463887	3.538948	-0.490500
H	-2.123318	3.594402	-1.675878
H	-3.766986	3.171969	-2.209626
C	-3.825028	1.217613	2.431422
H	-4.864599	1.401421	2.698385
H	-3.298415	2.177355	2.383845
H	-3.359399	0.588648	3.198429
C	-4.529072	-2.469093	0.484612

Chlorodimethylallylsilane: Rhamnose-TS-α-$^3S_1$
***E* =** -1601.027657
***H* =** -1600.605036
***qh-G* =** -1600.65207
N_{imag} = 1

Cl	4.321006	0.046900	1.707011
H	-5.418843	1.941537	-1.511012
H	-3.789165	-2.871760	-1.728957
H	-5.633478	1.596014	0.222547
C	0.666203	-0.251289	0.502602
H	0.820880	0.600382	1.155053
H	0.096746	-1.076451	0.914965
C	1.530103	-0.452735	-0.540182
H	1.419487	-1.365847	-1.121384
C	2.572524	0.475802	-0.969781
H	2.420103	1.486749	-0.581766
H	2.655454	0.504846	-2.062295
Si	4.344618	-0.046874	-0.403994
C	5.557496	1.223136	-1.030351

H	5.298255	2.228182	-0.690628
H	5.569845	1.218823	-2.124276
H	6.564808	0.988340	-0.677135
C	4.742039	-1.799956	-0.900056
H	5.753582	-2.061740	-0.579290
H	-3.881580	-1.185574	-2.278822
H	4.047588	-2.517148	-0.457180
O	-1.418282	-0.538053	-1.370905
C	-3.065756	-1.125919	0.421300
C	-1.857568	1.050022	0.484600
C	-3.255036	0.395852	0.432728
C	-0.984238	0.446646	-0.634869
C	-2.322412	-1.588752	-0.845407
H	-0.444307	1.176886	-1.228651
H	-1.647671	-2.404540	-0.575874
C	-3.203328	-1.994478	-2.011612
H	-2.588368	-2.255945	-2.874152
H	-1.438405	0.853995	1.472558
O	-1.980686	2.428756	0.236448
C	-1.008171	3.255353	0.881804
H	-1.002762	3.077220	1.962241
H	-1.300080	4.285042	0.683741
H	-0.002988	3.089805	0.479034
O	-2.332626	-1.446816	1.598468
H	-4.050550	-1.602980	0.440908
C	-2.583363	-2.755432	2.112469
H	-2.289172	-3.537365	1.403108
H	-3.643430	-2.876199	2.360544
H	-1.986002	-2.855000	3.017324
O	-3.973933	0.757105	-0.725908
H	-3.794725	0.679693	1.340837
C	-4.902001	1.833388	-0.558236
H	-4.387265	2.763395	-0.310898
H	4.695565	-1.899197	-1.988702

Chlorodimethylallylsilane: Rhamnose-TS-β-$^{1}C_{4}$

***E* =** -1601.02907

***H* =** -1600.606496

***qh-G* =** -1600.653397

N_{imag} = 1

C	0.944127	-0.352100	-1.002730
C	2.843576	0.539851	0.438515
C	2.519870	-1.961952	-0.018103
C	3.522815	-0.823325	0.178293
O	1.391093	-1.571289	-0.908543
C	1.879568	0.806469	-0.729532
H	3.616813	1.315508	0.435404
H	3.010903	-2.723998	-0.621011
H	0.256890	-0.221451	-1.830693
O	4.309448	-0.746862	-1.003915
H	2.502120	0.791852	-1.642219
Si	-4.325363	-0.328441	-0.258562
C	-5.526134	0.081597	-1.625223
H	-5.609561	-0.763007	-2.315520
H	-5.207258	0.960497	-2.189694
H	-6.517008	0.279767	-1.208836
Cl	-4.199111	1.344224	1.027407

H	5.684805	0.728921	-0.411982
H	6.135345	-0.319779	-1.774351
H	6.182471	-0.959220	-0.111524
C	-4.807571	-1.810727	0.765412
H	-4.115310	-1.976624	1.593767
H	-4.819512	-2.708134	0.139296
H	-5.809542	-1.674204	1.179907
C	-2.567516	-0.530811	-1.026966
H	-2.362380	0.406919	-1.550822
H	-2.701691	-1.341725	-1.752847
C	-1.537848	-0.863797	-0.042580
H	-1.508738	-1.896704	0.298381
C	-0.608296	0.006596	0.455373
H	-0.680824	1.063192	0.226212
H	-0.014081	-0.264480	1.316425
O	1.154148	2.006823	-0.652461
O	2.204919	0.494662	1.698631
H	4.144442	-1.074971	1.044348
C	1.953541	-2.581208	1.249457
H	1.522500	-1.835843	1.914524
H	2.764286	-3.084531	1.780913
H	1.202585	-3.330004	0.992973
C	1.798223	3.132694	-1.260979
H	1.114954	3.972147	-1.147732
H	1.976637	2.947830	-2.325180
H	2.745185	3.372109	-0.767681
C	2.177396	1.734440	2.414652
H	1.562594	2.476979	1.901228
H	3.192610	2.124108	2.547676
H	1.747680	1.516229	3.391336
C	5.649280	-0.294676	-0.800261

Chlorodimethylallylsilane: Rhamnose-TS-β-$^{1}S_{5}$[30]

***E* =** -1601.026319

***H* =** -1600.60391

***qh-G* =** -1600.650712

N_{imag} = 1

C	-1.164324	-0.875243	-1.460434
C	0.377288	-1.283236	0.096135
C	-2.679477	-0.025914	0.429070
C	-1.866004	1.429854	-1.542011
C	-2.128269	1.346126	-0.033912
O	-1.004224	0.303319	-1.983713
C	-2.408458	-1.126038	-0.639206
O	-2.093575	-0.315378	1.680936
H	-0.991382	2.678168	-3.058415
O	-3.024098	2.403429	0.239393
H	-3.205075	-1.011978	-1.398052
H	-0.769385	-1.678164	-2.068980
H	-3.766235	0.062285	0.532246
H	-2.805264	1.303796	-2.088031
H	-1.180021	1.507383	0.495691
O	-2.449276	-2.406820	-0.080576
Si	3.928961	0.087608	-0.054088
C	-3.059743	2.823898	1.604807
H	-2.431492	-3.570913	-1.831399
H	-4.049927	-3.170487	-1.185379

H	-3.007143	-4.340010	-0.335528
H	-3.123878	-2.049946	2.237668
H	-3.860128	-0.511113	2.773756
H	-2.358078	-1.092631	3.533218
C	-2.917225	-1.042480	2.599801
H	-3.494824	2.059052	2.255052
C	2.205680	0.115561	-0.914779
H	1.686192	0.989649	-0.513486
H	2.451211	0.284801	-1.970103
C	1.436549	-1.118598	-0.746300
H	1.731124	-1.962025	-1.367999
H	0.111444	-0.516777	0.812727
H	-0.032232	-2.269589	0.276107
C	-1.160854	2.693043	-1.980964
H	-1.787160	3.549936	-1.734998
H	-0.201408	2.798675	-1.469918
H	-3.684432	3.715431	1.633935
H	-2.054414	3.069766	1.964220
C	-3.018399	-3.422078	-0.919608
C	4.769985	1.724238	-0.361312
Cl	3.536482	-0.064365	2.018252
C	4.939082	-1.395582	-0.562760
H	5.107790	-1.376616	-1.643844
H	4.443436	-2.334483	-0.306856
H	5.913353	-1.377305	-0.067846
H	5.726745	1.762812	0.165719
H	4.155239	2.560612	-0.021502
H	4.964712	1.849649	-1.430528

Chlorodimethylallylsilane: Rhamnose-TS-β-$^1S_3$
***E* =** -1601.021916
***H* =** -1600.599796
***qh-G* =** -1600.647164
N_{imag} = 1

C	-0.776842	-0.423155	-0.838686
C	-3.165734	-0.351552	-0.633548
C	-1.705002	1.670793	0.004073
C	-3.113087	1.026648	0.048169
O	-0.735122	0.856524	-0.812332
C	-1.910012	-1.163175	-0.172240
O	-4.359325	-0.992256	-0.253539
H	-0.110007	2.460636	1.224561
O	-3.984351	1.952281	-0.579070
H	-1.930237	-2.186225	-0.558022
H	-0.284272	-0.856949	-1.699178
H	-3.140057	-0.206057	-1.722077
H	-1.778454	2.558148	-0.619686
H	-3.401117	0.884075	1.093722
O	-1.789835	-1.145866	1.233804
Si	4.463668	0.371262	0.147317
Cl	5.416178	-0.944236	-1.216880
H	-2.089189	-3.194189	1.509247
H	-3.521357	-2.168608	1.819776
H	-2.143531	-2.153719	2.954792
C	5.229487	2.060585	-0.060094
H	4.747650	2.773500	0.615988
H	6.294748	2.030237	0.182164

H	5.118165	2.427001	-1.083109
C	4.665279	-0.344615	1.858547
H	4.168900	0.300325	2.590279
H	4.231064	-1.344071	1.931427
H	5.723158	-0.407416	2.125271
C	2.624722	0.436659	-0.385895
H	2.146111	1.153297	0.288870
H	2.632553	0.856919	-1.397799
C	1.946442	-0.873251	-0.367898
H	2.211923	-1.560395	-1.168646
C	1.034829	-1.283861	0.545944
H	0.777154	-0.677602	1.405284
H	0.675623	-2.305322	0.541677
C	-1.091278	2.000560	1.348214
H	-1.742590	2.723486	1.845349
H	-1.013092	1.113587	1.976325
C	-5.305623	2.015320	-0.033476
H	-5.859024	1.090385	-0.207184
H	-5.802995	2.842093	-0.539451
H	-5.272115	2.220379	1.042891
C	-4.866219	-1.929783	-1.207971
H	-5.053100	-1.440480	-2.169526
H	-5.804053	-2.305212	-0.802394
H	-4.180428	-2.770457	-1.355633
C	-2.436754	-2.235918	1.909646

Mannuronic Acid-R-$^{4}H_{3}$-like

***E* =** -842.594427

***H* =** -842.309624

***qh-G* =** -842.34839

N_{imag} = 0

C	-0.465021	-1.969961	-0.788143
O	-2.465890	1.063252	0.063628
C	-1.452145	0.366025	-0.617542
C	1.026153	-0.068060	-0.649422
C	-0.101014	0.736461	-0.004338
O	0.714338	-1.532681	-0.780483
C	-1.668879	-1.146395	-0.481499
H	-1.437823	0.624726	-1.684518
H	1.204232	0.255256	-1.674315
O	-1.702246	-1.535918	0.894323
H	-0.539132	-3.049407	-0.898802
O	0.237377	2.077371	-0.277902
H	-0.124811	0.537262	1.070061
C	2.337443	-0.023502	0.151643
H	-2.718856	-2.495843	2.362601
C	-3.520423	1.556571	-0.770038
H	-4.221784	2.061499	-0.108199
O	2.403815	0.349243	1.293960
H	0.456101	3.943475	0.482571
H	0.877322	2.642874	1.627916
H	-0.840533	2.995065	1.257762
O	3.340450	-0.453829	-0.598072
H	-4.040458	0.744655	-1.289136
H	-3.133212	2.270173	-1.504178
C	0.173436	2.959429	0.853038
C	4.647804	-0.508838	0.034563

H	4.616483	-1.186373	0.886957
H	4.940993	0.488720	0.358773
H	5.318818	-0.881042	-0.733332
C	-2.865490	-2.285098	1.306025
H	-3.758231	-1.673957	1.159165
H	-2.949870	-3.221281	0.750376
H	-2.543177	-1.495094	-1.033065

Mannuronic Acid-R-$^{3}H_{4}$-like
***E* =** -842.601235
***H* =** -842.317421
***qh-G* =** -842.35635
N_{imag} = 0

C	-0.997531	-0.462333	-1.701685
C	-0.841586	0.080535	0.722222
C	1.003501	0.628825	-0.900314
C	0.181612	1.146572	0.280789
O	0.176810	-0.082230	-1.917692
C	-1.781412	-0.155401	-0.483749
H	-1.429059	0.447053	1.569427
H	1.404859	1.473386	-1.458617
H	-1.443853	-1.041674	-2.509336
O	-0.469095	2.327681	-0.140042
H	-2.230562	0.828441	-0.727817
H	0.882643	1.335736	1.097784
C	2.142591	-0.350482	-0.568979
H	-0.885891	-1.398717	2.955095
H	0.036221	-2.727856	2.207773
C	-0.643376	3.314732	0.887151
O	2.276073	-1.430781	-1.072631
H	-1.269319	2.942358	1.703639
H	-1.136369	4.161468	0.414001
H	0.326646	3.630157	1.283204
O	-0.109385	-1.072855	1.046174
H	-4.057727	-0.143068	0.954863
C	-0.696189	-1.943929	2.024937
H	-1.622523	-2.382571	1.650952
O	2.955584	0.229724	0.304234
C	4.122907	-0.536783	0.707271
H	4.728257	-0.773796	-0.166337
H	3.806394	-1.452184	1.205243
H	4.663743	0.109930	1.391225
C	-4.056739	-0.694956	0.010727
H	-4.673163	-1.585290	0.111252
H	-4.452081	-0.063550	-0.790053
O	-2.736349	-1.161603	-0.315357

Chlorodimethylallylsilane: Mannuronic Acid-TS-α-$^{O}S_{2}$[30]
***E* =** -1789.631223
***H* =** -1789.192773
***qh-G* =** -1789.243765
N_{imag} = 1

C	0.863991	-0.947204	-0.713707
C	-0.988317	-1.735387	0.517993
C	3.187293	-0.669726	-0.137507
C	1.420210	1.140590	0.221193
C	2.909407	0.763477	0.361993

O	0.746103	0.336697	-0.797807
C	1.925085	-1.540414	0.168993
H	4.035889	-1.076034	0.422393
H	0.930208	0.940195	1.177793
H	3.517913	1.450371	-0.230407
O	2.026473	-2.898915	-0.152307
H	0.560486	-1.482702	-1.602507
O	3.459594	-0.605129	-1.519707
O	2.064631	3.439584	-0.092307
O	3.172508	0.897474	1.744493
H	1.698687	-1.389512	1.232793
Si	-4.385403	-0.227656	-0.211807
Cl	-4.416506	-0.557755	1.879093
H	2.570155	-4.734720	0.513293
H	2.050966	-3.622416	1.806793
H	3.660468	-3.436330	1.050893
C	-5.282916	-1.644747	-1.028607
H	-5.246515	-1.528948	-2.116207
H	-6.332416	-1.657138	-0.724207
H	-4.840125	-2.609051	-0.769807
C	-5.145087	1.444651	-0.539707
H	-5.122385	1.658951	-1.612507
H	-4.605080	2.238046	-0.018107
H	-6.187187	1.462061	-0.211007
C	-2.531202	-0.173173	-0.708807
H	-2.076695	0.644123	-0.142507
H	-2.553800	0.100727	-1.770307
C	-1.822214	-1.446180	-0.514407
H	-1.977421	-2.212478	-1.271007
H	-0.877710	-1.046688	1.348993
H	-0.588926	-2.734891	0.636293
C	1.190324	2.612792	-0.130407
O	-0.084174	2.838304	-0.424307
C	-0.436161	4.208108	-0.753807
H	-1.498562	4.183717	-0.976207
H	-0.231755	4.856806	0.096993
H	0.134442	4.538502	-1.620807
C	2.616165	-3.709121	0.873093
C	4.293484	-1.650236	-2.036807
C	4.514511	1.282862	2.056893
H	5.239704	0.529655	1.730793
H	4.561312	1.375461	3.140493
H	4.755420	2.245559	1.595393
H	4.512587	-1.381438	-3.069107
H	3.787075	-2.617032	-2.007507
H	5.230984	-1.707645	-1.472807

Chlorodimethylallylsilane: Mannuronic Acid-TS-α-$^{4}C_{1}$[30]

***E* =** -1789.630052

***H* =** -1789.190996

***qh-G* =** -1789.241989

N_{imag} = 1

C	0.625187	-0.871086	-1.158494
C	-1.022433	-1.659981	0.387622
C	2.422162	-1.111425	0.627548
C	1.321561	1.072102	0.079048
C	2.640561	0.389485	0.437179

O	0.591921	0.411617	-1.020913
C	1.791003	-1.695365	-0.649877
H	1.730989	-1.257144	1.467843
H	0.650777	1.063694	0.939046
O	2.698106	-1.544138	-1.732164
H	0.120384	-1.209173	-2.054833
O	3.036305	1.012584	1.641129
Si	-4.482244	-0.191703	-0.053789
C	-5.287748	1.452395	-0.412996
H	-5.395990	1.585426	-1.493621
H	-4.695885	2.282386	-0.020766
H	-6.283033	1.495294	0.036319
Cl	-4.257868	-0.365941	2.043782
H	4.567858	1.845177	2.675464
H	4.662765	2.099373	0.913442
H	5.060028	0.498888	1.613864
C	-5.461873	-1.664495	-0.647131
H	-4.987750	-2.607765	-0.367318
H	-5.555018	-1.634241	-1.737010
H	-6.468112	-1.648445	-0.221238
C	-2.699449	-0.177473	-0.771195
H	-2.194407	0.681530	-0.321305
H	-2.846391	0.017197	-1.839926
C	-1.963078	-1.435112	-0.566093
H	-2.204347	-2.254703	-1.239685
H	-0.636010	-2.658596	0.548624
H	-0.820972	-0.920278	1.155100
H	1.495203	-2.735913	-0.513893
O	3.664360	-1.713683	0.898840
H	3.369614	0.554919	-0.359941
C	1.517656	2.515933	-0.402725
O	2.553790	2.942801	-0.842648
O	0.383708	3.196653	-0.276568
C	0.400935	4.571891	-0.741256
H	0.631248	4.600101	-1.805735
H	1.144377	5.140313	-0.183859
H	-0.598824	4.950206	-0.550874
C	2.904695	-2.713338	-2.539896
H	3.294766	-3.531916	-1.929246
H	1.982448	-3.025823	-3.038157
H	3.640223	-2.430545	-3.290174
C	3.597522	-2.873573	1.730680
H	4.624608	-3.198687	1.888091
H	3.038513	-3.687160	1.255167
H	3.139630	-2.635765	2.697056
C	4.420457	1.380009	1.701736

Chlorodimethylallylsilane: Mannuronic Acid-TS-α-$^{3}S_{1}$

***E* =** -1789.630949

***H* =** -1789.191876

***qh-G* =** -1789.24278

N_{imag} = 1

Cl	5.112897	0.177806	1.455002
H	-4.777004	2.199102	-1.713698
H	-4.845004	2.650802	0.008302
C	1.267297	0.177405	0.740102
H	1.576297	1.183705	1.001502

H	0.636098	-0.338296	1.453702
C	1.966798	-0.512495	-0.207998
H	1.683198	-1.545795	-0.396698
C	3.054197	0.028905	-1.025298
H	3.091897	1.121205	-1.000298
H	2.975698	-0.314095	-2.063998
Si	4.803798	-0.581994	-0.494098
C	6.068697	0.195507	-1.622098
H	5.987797	1.284807	-1.622498
H	5.928598	-0.162893	-2.646098
H	7.077597	-0.075593	-1.301198
C	4.888199	-2.442794	-0.409998
H	5.894499	-2.763893	-0.129798
O	-3.120802	-1.599097	-2.046298
H	4.184699	-2.849694	0.319602
O	-1.059402	-0.537396	-0.770098
C	-2.572802	-0.095597	1.176502
C	-1.134303	1.720304	0.273402
C	-2.590703	1.318703	0.590002
C	-0.468403	0.599204	-0.541398
C	-2.094102	-1.085497	0.097302
H	0.107797	0.924304	-1.399698
H	-1.640002	-1.954197	0.579802
C	-3.184302	-1.606997	-0.847598
O	-4.174902	-2.126398	-0.119998
C	-5.273901	-2.715198	-0.859598
H	-5.954901	-3.096899	-0.104698
H	-4.906601	-3.521398	-1.493598
H	-5.759902	-1.954599	-1.469898
H	-0.623803	1.877704	1.224802
O	-1.132104	2.879304	-0.520298
C	0.014896	3.722104	-0.366998
H	0.165096	3.983704	0.685402
H	-0.187405	4.622604	-0.943598
H	0.921296	3.247504	-0.758598
O	-1.695802	-0.085597	2.288902
H	-3.584902	-0.378298	1.473802
C	-2.000002	-1.055497	3.295602
H	-1.905702	-2.078697	2.916002
H	-3.013502	-0.905697	3.680702
H	-1.279202	-0.905797	4.097202
O	-3.395103	1.263603	-0.565398
H	-2.988503	2.019703	1.329002
C	-4.179204	2.431502	-0.833598
H	-3.541704	3.293202	-1.037198
H	4.655899	-2.869994	-1.390298

Chlorodimethylallylsilane: Mannuronic Acid-TS-β-$^{1}C_{4}$[30]

***E* =** -1789.635464

***H* =** -1789.197031

***qh-G* =** -1789.248008

N_{imag} = 1

C	-0.679613	-0.776529	-1.108429
C	-2.622687	-0.941321	0.514665
C	-2.326383	1.001308	-1.069088
C	-3.336149	0.038984	-0.438443
O	-1.148461	0.324724	-1.611575

C	-1.566727	-1.697205	-0.310465
H	-3.351501	-1.660306	0.902272
H	-2.788922	1.443045	-1.951620
H	0.090181	-1.228009	-1.720740
O	-3.965211	-0.656682	-1.499550
H	-2.125377	-2.195872	-1.125747
Si	4.580895	0.217626	-0.427047
C	5.897910	-0.626963	-1.443840
H	5.863696	-0.260009	-2.473965
H	5.756202	-1.709925	-1.459134
H	6.890312	-0.413797	-1.039120
Cl	4.680023	-0.561147	1.539287
H	-5.408835	-1.720041	-0.403518
H	-5.684181	-1.514665	-2.148289
H	-5.934349	-0.137180	-1.045393
C	4.777310	2.069733	-0.316415
H	4.030790	2.519706	0.341605
H	4.674387	2.515089	-1.310484
H	5.768718	2.323496	0.067129
C	2.859296	-0.291003	-1.109486
H	2.828404	-1.383552	-1.071465
H	2.892928	0.034585	-2.156660
C	1.731550	0.328620	-0.398493
H	1.518733	1.371331	-0.619139
C	0.925418	-0.294742	0.502913
H	1.111113	-1.318915	0.800698
H	0.221240	0.280376	1.086977
O	-0.792017	-2.627324	0.395474
O	-2.065965	-0.173768	1.559967
H	-4.051937	0.638182	0.129883
C	-1.848826	2.147835	-0.169560
O	-0.695634	2.421214	0.040478
O	-2.902450	2.824355	0.275304
C	-2.625253	3.977299	1.112234
H	-2.008324	4.691493	0.568581
H	-2.116052	3.658812	2.021013
H	-3.597768	4.401709	1.342639
C	-1.342145	-3.950756	0.441337
H	-0.620300	-4.559465	0.982010
H	-1.473619	-4.350671	-0.569067
H	-2.298703	-3.972634	0.972259
C	-2.050464	-0.800180	2.848484
H	-1.406887	-1.682723	2.850551
H	-3.065158	-1.078926	3.151711
H	-1.658324	-0.060445	3.544472
C	-5.325151	-1.024766	-1.244990

Chlorodimethylallylsilane: Mannuronic Acid-TS-β-$^{1}S_{5}$[30]

***E* =** -1789.628191

***H* =** -1789.189359

***qh-G* =** -1789.2402

N_{imag} = 1

C	-1.015842	-0.878617	-1.596035
C	0.650318	-1.759573	-0.236142
C	-2.520790	-0.922165	0.489157
C	-1.806718	1.203517	-0.758797
C	-2.015447	0.533653	0.602821

O	-0.915930	0.416657	-1.616939
C	-2.212885	-1.505616	-0.927359
O	-1.915872	-1.654960	1.528862
O	-0.001614	2.679542	-0.157552
O	-2.912215	1.385866	1.278405
H	-3.038021	-1.159525	-1.579760
H	-0.565755	-1.362393	-2.451685
H	-3.609507	-0.916171	0.606878
H	-2.749102	1.317079	-1.295186
H	-1.049109	0.507118	1.120045
O	-2.153329	-2.899671	-0.906116
Si	4.000048	-0.044257	-0.024898
C	-2.860109	1.303018	2.707504
H	-2.128096	-3.287071	-2.971980
H	-3.742233	-3.302988	-2.202527
H	-2.583489	-4.621111	-1.888488
H	-2.881168	-3.503311	1.350081
H	-3.663603	-2.328975	2.449705
H	-2.134118	-3.110418	2.921047
C	-2.705861	-2.715178	2.082741
H	-3.214074	0.333324	3.067518
C	-1.414912	4.894446	-0.788807
H	-2.204693	5.545527	-1.150925
H	-0.504724	5.024060	-1.372940
C	2.291623	0.030172	-0.901965
H	1.689966	0.745743	-0.334687
H	2.524509	0.459567	-1.883367
C	1.630629	-1.271182	-1.045902
H	1.946754	-1.886724	-1.886099
H	0.373007	-1.252201	0.678401
H	0.295468	-2.775879	-0.351186
C	-1.121412	2.560190	-0.589138
O	-1.924433	3.546193	-0.961729
H	-1.211918	5.079326	0.265452
H	-3.515037	2.088505	3.080455
H	-1.840466	1.472889	3.068612
C	-2.685374	-3.553092	-2.068538
C	4.705484	1.680974	0.050440
Cl	3.610407	-0.675363	1.957219
C	5.135409	-1.300140	-0.809589
H	5.326993	-1.028298	-1.852131
H	4.703806	-2.303286	-0.788643
H	6.093856	-1.328480	-0.285368
H	5.644001	1.685341	0.610317
H	4.011676	2.373695	0.531764
H	4.909944	2.043957	-0.961271

Chlorodimethylallylsilane: Mannuronic Acid-TS-β-$^{1}S_{3}$[30]

***E* =** -1789.628535

***H* =** -1789.189997

***qh-G* =** -1789.240817

N_{imag} = 1

C	-0.592850	-1.255825	-0.544820
C	-2.976638	-1.118192	-0.417183
C	-1.509989	0.911621	-0.964013
C	-2.950826	0.389567	-0.740614
O	-0.562608	-0.168743	-1.256457

C	-1.711324	-1.451993	0.444063
O	-4.165982	-1.405469	0.277040
O	0.224318	1.813813	0.470982
O	-3.667626	0.685101	-1.925414
H	-1.715858	-2.492932	0.777416
H	-0.192071	-2.115239	-1.065845
H	-2.941497	-1.675093	-1.362560
H	-1.522065	1.478138	-1.894522
H	-3.382063	0.922127	0.109522
O	-1.603339	-0.556670	1.527784
Si	4.439114	0.207522	-0.168416
Cl	5.605841	-1.528721	-0.519790
H	-1.893713	-1.965220	3.040112
H	-3.338792	-0.999114	2.615440
H	-1.979547	-0.244575	3.493364
C	5.118452	1.576066	-1.238728
H	4.531334	2.487585	-1.092542
H	6.155128	1.791856	-0.968209
H	5.085603	1.310146	-2.297547
C	4.502629	0.573462	1.659555
H	3.888220	1.451176	1.882260
H	4.132077	-0.264922	2.253181
H	5.528149	0.788727	1.969877
C	2.659970	-0.217073	-0.745441
H	2.083771	0.699816	-0.587045
H	2.743939	-0.423540	-1.817229
C	2.063188	-1.351918	-0.029801
H	2.349818	-2.345982	-0.366463
C	1.190349	-1.255139	1.012328
H	0.928778	-0.295870	1.437417
H	0.891399	-2.145028	1.553163
C	-0.926336	1.832899	0.112873
O	-1.845032	2.703742	0.508697
C	-1.415634	3.715647	1.457193
H	-2.288385	4.340632	1.619959
H	-1.100218	3.240312	2.385093
H	-0.595321	4.294675	1.035294
C	-5.074007	0.881967	-1.749817
H	-5.584611	-0.045960	-1.482299
H	-5.450879	1.243750	-2.705605
H	-5.266055	1.634515	-0.976631
C	-4.650776	-2.741351	0.107179
H	-4.828934	-2.955079	-0.951873
H	-5.590355	-2.798645	0.653611
H	-3.955357	-3.482203	0.514869
C	-2.255746	-0.977289	2.737392

General experimental procedures

All chemicals (Acros, Fluka, Merck, and Sigma-Aldrich) were used as received unless stated otherwise. Dichloromethane was stored over activated 4 Å molecular sieves (beads, 8-12 mesh, Sigma-Aldrich). Before use traces of water present in the donor, diphenyl sulfoxide (Ph_2SO) and tri-*tert*-butylpyrimidine (TTBP) were removed by co-evaporation with dry toluene. The acceptors were stored in stock solutions (DCM, 0.50 M) over activated 3 Å molecular sieves (rods, size 1/16 in., Sigma-Aldrich). Trifluoromethanesulfonic anhydride (Tf_2O) was distilled over P_2O_5 and stored at –20 °C under a nitrogen atmosphere. Overnight temperature control was achieved by an FT902 Immersion Cooler (Julabo). Column chromatography was performed on silica gel 60 Å (0.04 – 0.063 mm, Screening Devices B.V.). TLC-analysis was conducted on TLC Silica gel 60 (Kieselgel 60 F_{254}, Merck) with UV detection by (254 nm) and by spraying with 20% sulfuric acid in ethanol followed by charring at ±150 °C or by spraying with a solution of $(NH_4)_6Mo_7O_{24}\cdot H_2O$ (25 g/l) and $(NH_4)_4Ce(SO_4)_4\cdot 2H_2O$ (10 g/l) in 10% sulfuric acid in water followed by charring at ± 250 °C. High-resolution mass spectra were recorded on a Thermo Finnigan LTQ Orbitrap mass spectrometer equipped with an electrospray ion source in positive mode (source voltage 3.5 kV, sheath gas flow 10, capillary temperature 275 °C) with resolution R=60.000 at m/z=400 (mass range = 150-4000). ^{1}H and ^{13}C NMR spectra were recorded on a Bruker AV-400 NMR instrument (400 and 101 MHz respectively), a Bruker AV-500 NMR instrument (500 and 126 MHz respectively), or a Bruker AV-600 NMR instrument (600 and 151 MHz respectively). For samples measured in $CDCl_3$ chemical shifts (δ) are given in ppm relative to tetramethylsilane as an internal standard or the residual signal of the deuterated solvent. Coupling constants (*J*) are given in Hz. To get better resolution of signals with small coupling constants or overlapping signals a gaussian window function (LB ± -1 and GB ± 0.5) was used on the ^{1}H NMR spectrum. All given ^{13}C-APT spectra are proton decoupled. NMR peak assignment was made using HH-COSY, HSQC. If necessary additional HH-NOESY, HMBC and HMBC-GATED experiments were used to elucidate the structure further. The anomeric product ratios were based on the integration of ^{1}H.

General glycosylation procedure: pre-activation Tf_2O/Ph_2SO based *C*-glycosylation.

A solution of the glycosyl donor (100 µmol), Ph_2SO (26 mg, 130 µmol, 1.3 equiv.) and TTBP (62 mg, 250 µmol, 2.5 equiv.) in DCM (2.0 mL, 0.050 M) was stirred over activated 3 Å molecular sieves (rods, size 1/16 in., Sigma-Aldrich) for 30 min under an atmosphere of N_2. The solution was cooled to −80 °C and Tf_2O (22.0 µL, 130 µmol, 1.3 equiv.) was slowly added to the reaction mixture. The reaction mixture was allowed to warm to −60 °C in approximately 45 min, followed by cooling to −80 °C and the addition of the acceptor (200.0 µmol, 2.0 equiv.) in DCM (0.40 mL, 0.50 M). The reaction was allowed to warm up to −60 °C and stirred for an additional 18 hours at this temperature until full reaction completion was observed. The reaction was quenched with sat. aq. $NaHCO_3$ at −60 °C and diluted with DCM (5 mL). The resulting solution was washed with H_2O and brine, dried over $MgSO_4$, filtered and concentrated under reduced pressure. Purification by column chromatography yielded the corresponding *C*-coupled glycoside. The anomeric ratio of both the crude product and the purified product were determined from ^{1}H-NMR and subsequently compared to ensure no alteration of the anomeric ratio as a result of column chromatography.

Synthetic procedures of glycosylation products

1-Allyl-1-deoxy-2,3,4,6-tetra-*O*-benzyl-α-D-glucopyranoside (S1)

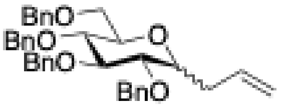

The title compound was prepared according to the general glycosylation procedure, using glucose donor **13**[32] and allyl(chloro)dimethylsilane (32 µmol, 18 mg, 32%, >98:2, α:β), allyltrimethylsilane (41 µmol, 23 mg, 41%, >98:2, α:β), or allyltributylstannane (70 µmol, 40 mg, 70%, <2:98, α:β), yielding the title compound as a colorless oil. Data for the α-anomer: ^{1}H NMR (400 MHz, $CDCl_3$, HH-COSY, HSQC, HMBC) δ 7.36 – 7.24 (m, 20H, CH_{arom}), 5.81 (ddt, *J* = 17.2, 10.2, 6.8 Hz, 1H, $CH_2CH{=}CH_2$ Allyl), 5.14 – 5.03 (m, 2H, $CH_2CH{=}CH_2$ Allyl), 4.93 (d, *J* = 10.9 Hz, 1H, C*H*H Bn), 4.84 – 4.78 (m, 2H, C*H*H Bn, CH*H* Bn), 4.72 – 4.59 (m, 3H, C*H*H Bn, C*H*H Bn, CH*H* Bn), 4.51 – 4.41 (m, 2H, CH*H* Bn, CH*H* Bn), 4.13 (dt, *J* = 10.2, 5.1 Hz, 1H, H-1), 3.81 – 3.74 (m, 2H, H-2 H-3), 3.73 – 3.66 (m, 1H, H-6), 3.62 (td, *J* = 7.3, 3.5 Hz, 3H, H-4, H-5, H-6), 2.56 – 2.40 (m, 2H, $CH_2CH{=}CH_2$ Allyl); ^{13}C-APT NMR (101 MHz, $CDCl_3$, HSQC, HMBC) δ 138.9,

138.3, 138.29, 138.2 (C_q), 134.9 ($CH_2CH=CH_2$ Allyl), 128.6, 128.6, 128.5, 128.3, 128.1, 128.1, 128.1, 128.0, 127.9, 127.1, 127.9, 127.8 (CH_{arom}), 117.0 ($CH_2CH=CH_2$ Allyl), 82.5 (C-3), 80.2 (C-2), 78.2 (C-4), 75.6 (CH_2 Bn), 75.2 (CH_2 Bn), 73.8 (C-1), 73.6 (CH_2 Bn), 73.2 (CH_2 Bn), 71.2 (C-5), 69.0 (C-6), 29.9 ($CH_2\mathit{CH}=CH_2$ Allyl); diagnostic signals for the β-anomer: ^{1}H NMR (400 MHz, $CDCl_3$, HH-COSY, HSQC, HMBC) δ 5.99 – 5.88 (m, 1H, $CH_2CH=CH_2$ Allyl), 3.37 – 3.32 (m, 1H, H-1), 2.60 (dddd, *J* = 12.2, 6.1, 3.0, 1.4 Hz, 1H, C*H*HCH=CH_2 Allyl), 2.32 (dt, *J* = 14.2, 6.6 Hz, 1H, CH*H*CH=CH_2 Allyl); ^{13}C-APT NMR (101 MHz, $CDCl_3$, HSQC, HMBC) δ 134.9 ($CH_2CH=CH_2$ Allyl), 117.1 ($CH_2CH=CH_2$ Allyl), 87.4 (C-3), 81.7 (C-4), 79.1 (C-2), 78.7 (C-1), 75.7 (CH_2 Bn), 75.1 (CH_2 Bn), 69.1 (C-6), 36.1 ($CH_2CH=CH_2$ Allyl); HRMS (ESI) [M/Z]: $[M + NH_4]^+$ calcd. for $C_{37}NH_{44}O_5^+$ 582.3214, found 582.3217.

1-Allyl-1-deoxy-2,3,4,6-tetra-*O*-benzyl-ᴅ-mannopyranoside (S2)

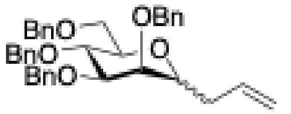

The title compound was prepared according to the general glycosylation procedure, using mannose donor **14**[32] and allyl(chloro)dimethylsilane (23 µmol, 13 mg, 23%, >98:2, α:β), allyltrimethylsilane (66 µmol, 37 mg, 66%, 72:28, α:β), or allyltributylstannane (69 µmol, 38 mg, 69%, 28:72, α:β), affording the title compound as a colorless oil. Data for the α-anomer: ^{1}H NMR (400 MHz, $CDCl_3$, HH-COSY, HH-NOESY, HSQC, HMBC) δ 7.41 – 7.23 (m, 20H, CH_{arom}), 5.81 – 5.69 (m, 1H, $CH_2C\mathit{H}=CH_2$ Allyl), 5.05 – 5.02 (m, 2H, $CH_2CH=CH_2$ Allyl), 4.72 (t, *J* = 5.9 Hz, 1H, CH_2 Bn), 4.63 – 4.50 (m, 7H, CH_2 Bn), 4.09 – 4.01 (m, 1H, H-1), 3.85 (m, *J* = 6.1 Hz, 2H, H-4, H-5), 3.80 – 3.75 (m, 2H, H-6 H-3), 3.73 – 3.68 (m, 1H, H-6), 3.62 (dd, *J* = 4.6, 3.0 Hz, 1H, H-2), 2.39 – 2.26 (m, 2H, $CH_2CH=CH_2$ Allyl); ^{13}C-APT NMR (101 MHz, $CDCl_3$, HSQC, HMBC) δ 134.5 ($CH_2\mathit{CH}=CH_2$ Allyl), 128.5, 128.5, 128.5, 128.4, 128.2, 128.1, 128.0, 127.9, 127.8, 127.8 (CH_{arom}), 117.3 ($CH_2\mathit{C}H=CH_2$ Allyl), 77.0 (C-3), 75.3 (C-2), 75.0 (C-4), 74.0 (CH_2 Bn), 73.8 (C-5), 73.4 (CH_2 Bn), 72.5 (C-1), 72.2 (CH_2 Bn), 71.6 (CH_2 Bn), 69.3 (C-6), 29.8 ($\mathit{CH_2}CH=CH_2$ Allyl); Diagnostic signals for the β-anomer: ^{1}H NMR (400 MHz, $CDCl_3$, HH-COSY, HSQC, HMBC) δ 7.54 – 7.16 (m, 20H, CH_{arom}), 5.81 – 5.62 (m, 1H, $CH_2C\mathit{H}=CH_2$ Allyl), 5.07 – 4.98 (m, 2H, $CH_2CH=CH_2$ Allyl), 4.87 (d, *J* = 10.7 Hz, 1H, C*H*H Bn), 4.81 – 4.71 (m, 2H, C*H*H Bn CH*H* Bn), 4.70 – 4.63 (m, 2H, C*H*H Bn, C*H*H Bn), 4.62 – 4.51 (m, 3H, CH*H* Bn, CH*H* Bn, CH*H* Bn), 3.95 – 3.85 (m, 1H, H-4), 3.79 (td, *J* = 3.3, 1.4 Hz, 1H, H-2), 3.76 (dd, *J* = 4.4, 1.9 Hz, 1H, H-6), 3.74 – 3.65 (m, 1H, H-6), 3.61 (dd, *J* = 9.5, 2.8 Hz, 1H, H-3), 3.46 (ddd, *J* = 9.7, 5.9, 1.9 Hz, 1H, H-5), 3.36 – 3.31 (m, 1H, H-1), 2.51 (dtt, *J* = 14.3, 6.4, 1.6 Hz, 1H, CH*H*CH=CH_2 Allyl), 2.38 – 2.25 (m, 1H, CH*H*CH=CH_2 Allyl); ^{13}C-APT NMR (101 MHz, $CDCl_3$, HSQC, HMBC) δ 138.9, 138.6, 138.4 (C_q), 134.8 ($\mathit{CH_2}CH=CH_2$ Allyl), 128.5, 128.5, 128.5, 128.4, 128.4, 128.1, 128.0, 127.8, 127.8, 127.6, 127.6 (CH_{arom}), 117.4 ($CH_2CH=\mathit{C}H_2$ Allyl), 85.6 (C-3), 80.0 (C-5), 78.4 (C-1), 75.6 (C-4), 75.4 (CH_2 Bn), 74.7 (C-2), 74.4, 73.7, 72.6, 72.5 (CH_2 Bn), 69.8 (C-6), 35.8 ($\mathit{CH_2}CH=CH_2$ Allyl); HRMS (ESI) [M/Z]: $[M + NH_4]^+$ calcd. for $C_{37}NH_{44}O_5^+$ 582.3214, found 582.3212.

1-Allyl-1-deoxy-2,3,4-tri-*O*-benzyl-ᴅ-rhamnopyranoside (S3)

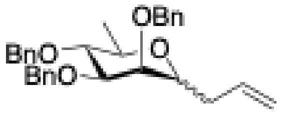

The title compound was prepared according to the general glycosylation procedure, using rhamnosyl donor **15**[33] and allyl(chloro)dimethylsilane (43 µmol, 20 mg, 43%, 89:11, α:β), allyltrimethylsilane (72 µmol, 33 mg, 72%, 50:50, α:β), or allyltributylstannane (77 µmol, 35 mg, 77%, 25:75, α:β), yielding the title compound as a colorless oil. Data for the α-anomer: ^{1}H NMR (400 MHz, $CDCl_3$, HH-COSY, HH-NOESY, HSQC, HMBC) δ 7.42 – 7.23 (m, 15H, CH_{arom}), 5.68 (ddt, *J* = 17.1, 10.2, 6.9 Hz, 1H, $CH_2C\mathit{H}=CH_2$ Allyl), 5.04 – 4.94 (m, 2H, $CH_2CH=C\mathit{H}H$ Allyl, $CH_2CH=CH\mathit{H}$ Allyl), 4.84 (d, *J* = 11.1 Hz, 1H, C*H*H Bn), 4.69 – 4.53 (m, 5H, CH*H* Bn, 2x CH_2 Bn), 4.01 (ddd, *J* = 8.1, 6.6, 3.4 Hz, 1H, H-1), 3.74 (dd, *J* = 7.9, 3.1 Hz, 1H, H-3), 3.66 (dd, *J* = 7.6, 6.2 Hz, 1H, H-5), 3.62 (t, *J* = 3.3 Hz, 1H, H-2), 3.58 (t, *J* = 7.8 Hz, 1H, H-4), 2.35 (dddt, *J* = 14.8, 8.2, 6.7, 1.4 Hz, 1H, C*H*HCH=CH_2 Allyl), 2.28 – 2.15 (m, 1H, CH*H*CH-CH_2 Allyl), 1.33 (d, *J* = 6.2 Hz, 3H, H-6); ^{13}C-APT NMR (101 MHz, $CDCl_3$, HSQC, HMBC) δ 138.6, 138.4, 138.4 (C_q), 134.3 ($CH_2\mathit{CH}=CH_2$ Allyl), 128.5, 128.5, 128.4, 128.2, 128.1, 128.0, 127.8, 127.8, 127.8 (CH_{arom}), 117.3 ($CH_2CH=\mathit{CH_2}$ Allyl), 80.3 (C-4), 78.1 (C-3), 75.2 (C-2), 74.8 (CH_2 Bn), 73.1 (C-1), 72.1 (CH_2 Bn), 71.8 (CH_2 Bn), 69.8 (C-5), 34.4 ($CH_2CH=CH_2$ Allyl), 18.2 (C-6); diagnostic signals for the β-anomer: ^{1}H NMR (400 MHz, $CDCl_3$, HH-COSY, HH-NOESY, HSQC, HMBC): δ 3.78 (dd, *J* = 2.7, 1.0 Hz, 1H, H-2), 3.36 – 3.28 (m, 2H, H-1, H-5), 2.50 – 2.39 (m, 1H, C*H*HCH=CH_2 Allyl); ^{13}C-APT NMR (101 MHz, $CDCl_3$, HSQC, HMBC): δ

134.9 ($CH_2CH=CH_2$ Allyl), 117.3 ($CH_2CH=CH_2$ Allyl), 78.3 (C-1), 76.1 (C-5), 74.9 (C-2), 35.9 ($CH_2CH=CH_2$ Allyl), 19.6 (C-6); HRMS (ESI) [M/Z]: [M + Na]$^+$ calcd. for $C_{30}H_{34}O_4Na^+$ 481.2349, found 481.2355.

Methyl (1-allyl-1-deoxy-2,3,4,6-tri-*O*-benzyl-α-ᴅ-mannopyranosyl uronate) (S4)

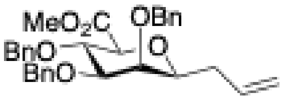

The title compound was prepared according to the general glycosylation procedure, using mannuronic acid donor **16**[33] and allyl(chloro)dimethylsilane (39 µmol, 20 mg, 39%, <2:98, α:β), allyltrimethylsilane (73 µmol, 37 mg, 73%, <2:98, α:β), or allyltributylstannane (64 µmol, 32 mg, 64%, <2:98, α:β), yielding the title compound as a colorless oil. Data for the β-anomer: ^{1}H NMR (600 MHz, $CDCl_3$, HH-COSY, HH-NOESY, HSQC, HMBC) δ 7.41 – 7.22 (m, 15H, CH_{arom}), 5.64 (dddd, *J* = 19.6, 9.4, 8.0, 6.1 Hz, 1H, $CH_2CH=CH_2$ Allyl), 5.03 (d, *J* = 11.6 Hz, 1H, C*H*H Bn), 5.02 (d, *J* = 1.3 Hz, 1H, $CH_2CH=CHH$ Allyl), 4.99 (dt, *J* = 5.0, 1.7 Hz, 1H, $CH_2CH=CHH$ Allyl), 4.86 (d, *J* = 10.7 Hz, 1H, C*H*H Bn), 4.78 (d, *J* = 11.8 Hz, 1H, C*H*H Bn), 4.75 (d, *J* = 11.8 Hz, 1H, CH*H* Bn), 4.69 (d, *J* = 11.7 Hz, 1H, CH*H* Bn), 4.66 (d, *J* = 10.7 Hz, 1H, CH*H* Bn), 4.23 (t, *J* = 9.6 Hz, 1H, H-4), 3.82 (d, *J* = 9.7 Hz, 1H, H-5), 3.78 (dd, *J* = 2.8, 1.0 Hz, 1H, H-2), 3.72 (s, 3H, CH_3 OMe), 3.61 (dd, *J* = 9.5, 2.7 Hz, 1H, H-3), 3.36 (td, *J* = 6.9, 1.1 Hz, 1H, H-1), 2.49 (dtt, *J* = 14.4, 6.3, 1.5 Hz, 1H, $CHHCH=CH_2$ Allyl), 2.31 (dtt, *J* = 14.0, 7.0, 1.1 Hz, 1H, $CHHCH=CH_2$ Allyl). ^{13}C-APT NMR (151 MHz, $CDCl_3$, HSQC, HMBC) δ 170.3 (C-6), 139.6, 139.2, 139.2 (C_q), 135.1 ($CH_2CH=CH_2$ Allyl), 129.5, 129.4, 129.3, 129.2, 129.1, 128.8, 128.7, 128.6, 128.5 (CH_{arom}), 118.6 ($CH_2CH=CH_2$ Allyl), 85.6 (C-3), 80.4 (C-1), 80.2 (C-5), 77.4 (C-4), 76.3 (CH_2 Bn), 75.4 (CH_2 Bn), 75.4 (C-2), 73.7 (CH_2 Bn), 53.4 (CH_3 OMe), 36.4 ($CH_2CH=CH_2$ Allyl). HRMS (ESI) [m/z]: [M + Na]$^+$ calcd. for $C_{31}H_{34}NaO_6^+$ 525.2248 found, 525.2253.

References

(1) Frisch, M. J.; Trucks, G. W.; Cheeseman, J. R.; Scalmani, G.; Caricato, M.; Hratchian, H. P.; Li, X.; Barone, V.; Bloino, J.; Zheng, G.; Vreven, T.; Montgomery, J. A.; Petersson, G. A.; Scuseria, G. E.; Schlegel, H. B.; Nakatsuji, H.; Izmaylov, A. F.; Martin, R. L.; Sonnenberg, J. L.; Peralta, J. E.; Heyd, J. J.; Brothers, E.; Ogliaro, F.; Bearpark, M.; Robb, M. A.; Mennucci, B.; Kudin, K. N.; Staroverov, V. N.; Kobayashi, R.; Normand, J.; Rendell, A.; Gomperts, R.; Zakrzewski, V. G.; Hada, M.; Ehara, M.; Toyota, K.; Fukuda, R.; Hasegawa, J.; Ishida, M.; Nakajima, T.; Honda, Y.; Kitao, O.; Nakai, H. Gaussian 09 Rev. D.01, 2009.

(2) Becke, A. D. Density-functional Thermochemistry. III. The Role of Exact Exchange. *J. Chem. Phys.* **1993**, *98* (7), 5648–5652. https://doi.org/10.1063/1.464913.

(3) Lee, C.; Yang, W.; Parr, R. G. Development of the Colle-Salvetti Correlation-Energy Formula into a Functional of the Electron Density. *Phys. Rev. B Condens. Matter* **1988**, *37* (2), 785–789. https://doi.org/10.1103/physrevb.37.785.

(4) Vosko, S. H.; Wilk, L.; Nusair, M. Accurate Spin-Dependent Electron Liquid Correlation Energies for Local Spin Density Calculations: A Critical Analysis. *Can. J. Phys.* **1980**, *58* (8), 1200–1211. https://doi.org/10.1139/p80-159.

(5) Ditchfield, R.; Hehre, W. J.; Pople, J. A. Self-Consistent Molecular-Orbital Methods. IX. An Extended Gaussian-Type Basis for Molecular-Orbital Studies of Organic Molecules. *J. Chem. Phys.* **1971**, *54* (2), 724–728. https://doi.org/10.1063/1.1674902.

(6) Bootsma, A. N.; Wheeler, S. Popular Integration Grids Can Result in Large Errors in DFT-Computed Free Energies. ChemRxiv July 29, 2019. https://doi.org/10.26434/chemrxiv.8864204.v5.

(7) Remmerswaal, W. A.; Hansen, T.; Hamlin, T. A.; Codée, J. D. C. Origin of Stereoselectivity in S_E2' Reactions of Six-Membered Ring Oxocarbenium Ions. *Chem. Eur. J.* **2023**, *29* (14), e202203490. https://doi.org/10.1002/chem.202203490.

(8) Sun, X.; Soini, T. M.; Poater, J.; Hamlin, T. A.; Bickelhaupt, F. M. PyFrag 2019—Automating the Exploration and Analysis of Reaction Mechanisms. *J. Comput. Chem.* **2019**, *40* (25), 2227–2233. https://doi.org/10.1002/jcc.25871.

(9) Ribeiro, R. F.; Marenich, A. V.; Cramer, C. J.; Truhlar, D. G. Use of Solution-Phase Vibrational Frequencies in Continuum Models for the Free Energy of Solvation. *J. Phys. Chem. B* **2011**, *115* (49), 14556–14562. https://doi.org/10.1021/jp205508z.

(10) Luchini, G.; Alegre-Requena, J. V.; Funes-Ardoiz, I.; Paton, R. S. GoodVibes: Automated Thermochemistry for Heterogeneous Computational Chemistry Data. *F1000Res* **2020**, *9*, 291. https://doi.org/10.12688/f1000research.22758.1.

(11) Bickelhaupt, F. Understanding Reactivity with Kohn–Sham Molecular Orbital Theory: E2–S_N2 Mechanistic Spectrum and Other Concepts. *J. Comput. Chem.* **1999**, *20*, 114–128.

(12) Vermeeren, P.; van der Lubbe, S. C. C.; Fonseca Guerra, C.; Bickelhaupt, F. M.; Hamlin, T. A. Understanding Chemical Reactivity Using the Activation Strain Model. *Nat. Protoc.* **2020**, *15* (2), 649–667. https://doi.org/10.1038/s41596-019-0265-0.

(13) Bickelhaupt, F. M.; Houk, K. N. Analyzing Reaction Rates with the Distortion/Interaction-Activation Strain Model. *Angew. Chem. Int. Ed.* **2017**, *56* (34), 10070–10086. https://doi.org/10.1002/anie.201701486.

(14) Bickelhaupt, F. M.; Houk, K. N. Das Distortion/Interaction-Activation-Strain-Modell zur Analyse von Reaktionsgeschwindigkeiten. *Angew. Chem.* **2017**, *129* (34), 10204–10221. https://doi.org/10.1002/ange.201701486.

(15) Beek, B. van; Bochove, M. A. van; Hamlin, T. A.; Bickelhaupt, F. M. Nucleophilic Substitution at Di- and Triphosphates: Leaving Group Ability of Phosphate versus Diphosphate. *Electron. Struct.* **2019**, *1* (2), 024001. https://doi.org/10.1088/2516-1075/ab0af3.

(16) Vermeeren, P.; Hansen, T.; Jansen, P.; Swart, M.; Hamlin, T. A.; Bickelhaupt, F. M. A Unified Framework for Understanding Nucleophilicity and Protophilicity in the S_N2/E2 Competition. *Chem. Eur. J.* **2020**, *26* (67), 15538–15548. https://doi.org/10.1002/chem.202003831.

(17) Galabov, B.; Koleva, G.; Schaefer III, H. F.; Allen, W. D. Nucleophilic Influences and Origin of the S_N2 Allylic Effect. *Chem. Eur. J.* **2018**, *24* (45), 11637–11648. https://doi.org/10.1002/chem.201801187.

(18) Hansen, T.; Vermeeren, P.; Yoshisada, R.; Filippov, D. V.; van der Marel, G. A.; Codée, J. D. C.; Hamlin, T. A. How Lewis Acids Catalyze Ring-Openings of Cyclohexene Oxide. *J. Org. Chem.* **2021**, *86* (4), 3565–3573. https://doi.org/10.1021/acs.joc.0c02955.

(19) Hansen, T.; Vermeeren, P.; Bickelhaupt, F. M.; Hamlin, T. A. Origin of the α-Effect in S_N2 Reactions. *Angew. Chem. Int. Ed.* **2021**, *60* (38), 20840–20848. https://doi.org/10.1002/anie.202106053.

(20) Hansen, T.; Roozee, J. C.; Bickelhaupt, F. M.; Hamlin, T. A. How Solvation Influences the S_N2 versus E2 Competition. *J. Org. Chem.* **2022**, *87* (3), 1805–1813. https://doi.org/10.1021/acs.joc.1c02354.

(21) Bickelhaupt, F. M.; Baerends, E. J. Kohn-Sham Density Functional Theory: Predicting and Understanding Chemistry. In *Reviews in Computational Chemistry*; John Wiley & Sons, Ltd: New York, 2000; pp 1–86. https://doi.org/10.1002/9780470125922.ch1.

(22) Velde, G. te; Bickelhaupt, F. M.; Baerends, E. J.; Guerra, C. F.; Gisbergen, S. J. A. van; Snijders, J. G.; Ziegler, T. Chemistry with ADF. *Journal of Computational Chemistry* **2001**, *22* (9), 931–967. https://doi.org/10.1002/jcc.1056.

(23) Fonseca Guerra, C.; Snijders, J. G.; te Velde, G.; Baerends, E. J. Towards an Order-N DFT Method. *Theor Chem Acc* **1998**, *99* (6), 391–403. https://doi.org/10.1007/s002140050353.

(24) Baerends, E. J.; Ziegler, T.; Atkins, A. J.; Autschbach, J.; Bashford, D.; Baseggio, O.; Bérces, A.; Bickelhaupt, F. M.; Bo, C.; Boerritger, P. M.; Cavallo, L.; Daul, C.; Chong, D. P.; Chulhai, D. V.; Deng, L.; Dickson, R. M.; Dieterich, J. M.; Ellis, D. E.; van Faassen, M.; Ghysels, A.; Giammona, A.; van Gisbergen, S. J. A.; Goez, A.; Götz, A. W.; Gusarov, S.; Harris, F. E.; van den Hoek, P.; Hu, Z.; Jacob, C. R.; Jacobsen, H.; Jensen, L.; Joubert, L.; Kaminski, J. W.; van Kessel, G.; König, C.; Kootstra, F.; Kovalenko, A.; Krykunov, M.; van Lenthe, E.; McCormack, D. A.; Michalak, A.; Mitoraj, M.; Morton, S. M.; Neugebauer, J.; Nicu, V. P.; Noodleman, L.; Osinga, V. P.; Patchkovskii, S.; Pavanello, M.; Peeples, C. A.; Philipsen, P. H. T.; Post, D.; Pye, C. C.; Ramanantoanina, H.; Ramos, P.; Ravenek, W.; Rodríguez, J. I.; Ros, P.; Rüger, R.; Schipper, P. R. T.; Schlüns, D.; van Schoot, H.; Schreckenbach, G.; Seldenthuis, J. S.; Seth, M.; Snijders, J. G.; Solà, M.; M, S.; Swart, M.; Swerhone, D.; te Velde, G.; Tognetti, V.; Vernooijs, P.; Versluis, L.; Visscher, L.; Visser, O.; Wang, F.; Wesolowski, T. A.; van Wezenbeek, E. M.; Wiesenekker, G.; Wolff, S. K.; Woo, T. K.; Yakovlev, A. L. ADF2018, SCM, Theoretical Chemistry, Vrije Universiteit, Amsterdam, The Netherlands, https://www.scm.com.

(25) Van Lenthe, E.; Baerends, E. J. Optimized Slater-Type Basis Sets for the Elements 1–118. *J. Comput. Chem.* **2003**, *24* (9), 1142–1156. https://doi.org/10.1002/jcc.10255.

(26) Franchini, M.; Philipsen, P. H. T.; Visscher, L. The Becke Fuzzy Cells Integration Scheme in the Amsterdam Density Functional Program Suite. *J. Comput. Chem.* **2013**, *34* (21), 1819–1827. https://doi.org/10.1002/jcc.23323.

(27) Franchini, M.; Philipsen, P. H. T.; van Lenthe, E.; Visscher, L. Accurate Coulomb Potentials for Periodic and Molecular Systems through Density Fitting. *J. Chem. Theory Comput.* **2014**, *10* (5), 1994–2004. https://doi.org/10.1021/ct500172n.

(28) Lenthe, E. van; Baerends, E. J.; Snijders, J. G. Relativistic Regular Two-component Hamiltonians. *J. Chem. Phys.* **1993**, *99* (6), 4597–4610. https://doi.org/10.1063/1.466059.

(29) van Lenthe, E.; Baerends, E. J.; Snijders, J. G. Relativistic Total Energy Using Regular Approximations. *J. Chem. Phys.* **1994**, *101* (11), 9783–9792. https://doi.org/10.1063/1.467943.

(30) Some transition states for the glycosyl cations do not exist as stationary points on the PES. For the purpose of a comparison with the other reactions that do involve true saddle points, a constrained potential energy surface was constructed by stepwise scanning the C···Nucleophile bond distance. Instead of the non-existing TS, we use a TS-like geometry at the point on this constrained potential energy surface with a similar C···Nucleophile bond distance and C···Si bond stretch as the TS-α-$^4C_1$ was selected. Supplementary Figures S1 – S4 show how the associated increase in energy at this TS-like point compares with the barrier heights of the other reaction paths.

(31) Legault, C. Y. CYLview, 1.0b, Université de Sherbrooke, 2009. http://www.cylview.org.

(32) Hansen, T.; Lebedel, L.; Remmerswaal, W. A.; van der Vorm, S.; Wander, D. P. A.; Somers, M.; Overkleeft, H. S.; Filippov, D. V.; Désiré, J.; Mingot, A.; Bleriot, Y.; van der Marel, G. A.; Thibaudeau, S.; Codée, J. D. C. Defining the S_N1 Side of Glycosylation Reactions: Stereoselectivity of Glycopyranosyl Cations. *ACS Cent. Sci.* **2019**, *5* (5), 781–788. https://doi.org/10.1021/acscentsci.9b00042.

(33) Dinkelaar, J.; de Jong, A. R.; van Meer, R.; Somers, M.; Lodder, G.; Overkleeft, H. S.; Codée, J. D. C.; van der Marel, G. A. Stereodirecting Effect of the Pyranosyl C-5 Substituent in Glycosylation Reactions. *J. Org. Chem.* **2009**, *74* (14), 4982–4991. https://doi.org/10.1021/jo900662v.
